# Improving quality of care for persons with diabetes: an overview of systematic reviews - what does the evidence tell us?

**DOI:** 10.1186/2046-4053-2-26

**Published:** 2013-05-07

**Authors:** Julia Worswick, S Carolyn Wayne, Rachel Bennett, Michelle Fiander, Alain Mayhew, Michelle C Weir, Katrina J Sullivan, Jeremy M Grimshaw

**Affiliations:** 1 Cochrane Effective Practice and Organisation of Care Group, Centre for Practice-Changing Research, Ottawa Hospital Research Institute, The Ottawa Hospital – General Campus, 501 Smyth Road, Box 711, Ottawa, Ontario K1H 8M5, Canada; 2 Clinical Epidemiology Program, Ottawa Hospital Research Institute, Centre for Practice-Changing Research, Ottawa Hospital Research Institute, The Ottawa Hospital, General Campus, 501 Smyth Road, Box 711, Ottawa, Ontario K1H 8M5, Canada; 3 Department of Medicine, University of Ottawa, 451 Smyth Road, Ottawa, ON, K1H 8M5, Canada

**Keywords:** Diabetes mellitus, Quality assurance, Health care, Quality improvement, Evidence-based practice, Evidence-based medicine, Overview of systematic reviews, Diabetes management, Intervention strategies

## Abstract

**Background:**

Ensuring high quality care for persons with diabetes remains a challenge for healthcare systems globally with consistent evidence of suboptimal care and outcomes. There is increasing interest in quality improvement strategies to improve diabetes management as reflected by a growing number of systematic reviews. These reviews are of varying quality and dispersed across many sources. In this paper, we present an overview of systematic reviews evaluating the impact of interventions to improve the quality of diabetes care.

**Methods:**

We searched for systematic reviews evaluating the effectiveness of any intervention intended to improve intermediate patient outcomes and process of care measures for patients with any type of diabetes. Two reviewers independently screened search results, appraised each systematic review using AMSTAR and extracted data from high quality reviews (AMSTAR score ≥ 5). Within reviews, we used vote counting by direction of effect to report the number of studies favouring an intervention for each outcome. We produced summaries of results for each intervention category.

**Results:**

We identified 125 reviews of varying methodological quality and summarised key findings from 50 high quality reviews. We categorised reviews by quality improvement intervention. Eight reviews were broad based (involving a variety of strategies). Other reviews considered: patient education and support (n = 21), telemedicine (n = 10), provider role changes (n = 7), and organisational changes (n = 4). Reviews reported intermediate patient outcomes (e.g. glycaemic control) (n = 49) and process of care outcomes (n = 9). There was evidence of considerable overlap of included studies between reviews.

**Conclusions:**

There is consistent evidence from high quality systematic reviews that patient education and support, provider role changes, and telemedicine are associated with improvements in glycaemic and vascular risk factor control in patients. There is less evidence about the impact of quality improvement interventions on other key process measures such as screening patients for diabetic complications. This paper provides decision makers with a comprehensive overview of evidence from high quality systematic reviews about the effects of quality improvement interventions on improving diabetes care.

## Background

Diabetes is a complex health problem that results in significant morbidity and mortality and health care resource utilisation [1-3]. With projected increases in the incidence of diabetes worldwide, health systems continue to focus on improving and optimising diabetes care by influencing patient behaviour and improving efficiency of care [1-3]. Yet, providing high quality care for diabetics still remains a challenge for healthcare systems and providers.

Recognising that gaps exist between best and actual care, researchers, people with diabetes, clinicians and decision makers have shown an increasing interest in quality improvement (QI) strategies to improve diabetes management [4]. Quality improvement strategies are ‘multidisciplinary, systems-focused, data-driven methods of understanding and improving the efficiency, effectiveness, and reliability of health process and outcomes of care’. QI strategies attempt to ‘reduce the difference between health care processes or outcomes observed in practice and those potentially obtainable based on current evidence-based knowledge’ [5].

There is substantial evidence and consensus on what constitutes high quality diabetes care [6]. Despite this, suboptimal care and poor patient outcomes continue at the local, national and international level [7]. For example, In Canada, evidence-based clinical practice guidelines for the management of diabetes have been available since 1998 [8]. While these guidelines have contributed to improvements in care [8-10], Ontario diabetes patients are still not optimally managed [9]. For example, between 2005 and 2008 the proportion of diabetes patients who had annual eye exams or foot exams remained fairly static at approximately 51% [9]. Further, only 46% of elderly diabetic patients in Ontario filled prescriptions for both ACE inhibitors and statins despite recommendations that most diabetes patients should receive both [8,11,12].

The current interest in QI interventions to improve diabetes care has led to a profusion of primary studies on diabetes that is overwhelming. Whilst systematic reviews partly address this problem of information overload, decision makers often find it difficult to reliably retrieve and keep up to date with the growing volume of published systematic reviews [13]. In addition, available systematic reviews are of variable quality, complexity and length, and are published in a variety of sources [14]. Further synthesis of this evidence is needed to provide reliable and accessible information to clinicians and decision makers. Overviews of systematic reviews are an efficient way to gather the best available evidence in a single source to provide broad, cumulative statements that summarise the current evidence on the effectiveness of interventions. Such overviews are helpful as starting points for decision makers to unpack the evidence towards finding solutions to improving practice and identify areas where new research is needed. This paper reports an overview of systematic reviews of diabetes QI interventions.

## Methods

### Eligibility criteria

We included systematic reviews that evaluated interventions to improve the quality of diabetes care and management in patients of any age, with any type of diabetes, in any setting compared to usual care or other intervention(s). We defined a systematic review as a synthesis of research evidence in which literature searches, inclusion criteria, and critical appraisal methods were explicitly described. We excluded primary studies, and systematic reviews that addressed multiple chronic diseases where it was not possible to isolate the effects of QI interventions on diabetes care.

To be included, systematic reviews had to report effects on at least one intermediate patient outcome, (glycaemic control as measured by glycated haemoglobin (HbA1c) level. vascular risk factor control as measured by high-density lipoprotein, total cholesterol, or blood pressure levels, or maintaining smoking cessation for at least one year) or process of care measure (monitoring HbA1c levels in patients, prescribing appropriate medications such as acetylsalicylic acid, statins, or anti-hypertensive drugs to control vascular risks, conducting retinopathy screening or referring patients for eye examinations, performing foot examinations to screen patients for potential problems such as ulcers or infections, monitoring renal function through testing of creatinine or microalbumin, or prescribing nicotine replacement therapies to promote smoking cessation). We excluded systematic reviews that only reported changes in knowledge or attitudes towards treatment of diabetes [15].

### Search strategy to identify systematic reviews

We searched the following electronic databases without language restrictions: Medline, EMBASE, AARP Ageline, AMED (Allied and Complementary Medicine), and HealthSTAR via OVID, The Cochrane Database of Systematic Reviews and DARE (Database of Abstracts and Reviews) via Wiley, Health Systems Evidence (http://www.healthsystemsevidence.org/), Rx for Change (http://www.rxforchange.ca/), and Google.

We used two search strategies to identify potentially eligible systematic reviews. The strategies are based on those used by Shojania [16,17] and the Cochrane Effective Practice and Organisation of Care (EPOC) Group. Full details of the searches and rationale are provided in Additional file 1. Search dates spanned 1976 to April 2011.

### Selection

Two reviewers independently screened all titles and abstracts identified by the searches. We retrieved the full papers of citations that passed the initial screening, and two reviewers independently assessed each against the eligibility criteria. Reviewers compared results and resolved any discrepancies through discussion or third party adjudication.

### Quality assessment

Two reviewers independently assessed the methodological quality of all reviews that met the eligibility criteria using the ‘assessment of multiple systematic reviews’ (AMSTAR) checklist [18,19], an 11-item validated measurement tool where reviewers score one point for each criterion met, with higher scores indicating a higher level of methodological quality. Items on the AMSTAR checklist assess criteria such as the comprehensiveness of the search and whether the quality of included studies was evaluated and accounted for [20]. Reviewers compared scores for each item of the AMSTAR checklist and resolved disagreements through discussion or third party adjudication. To present the best available evidence, we extracted data from those systematic reviews that scored five or above [20].

### Data extraction

Two reviewers independently extracted data from each included systematic review using standard forms developed for this overview. We used a consensus process to ensure the consistency and reliability of the data and enlisted the assistance of a third party in cases of disagreement. For each included systematic review, we extracted review level information on the objectives, publication year, number of included studies, search dates, country of origin, method(s) of analysis used, as well as relevant quantitative data such as pooled effect sizes. We did not retrieve primary study publications; rather we abstracted primary study data reported within each systematic review on study design, population, intervention(s), comparator, and direction of effect, whether reported descriptively or numerically. We classified the interventions used in each study using the McMaster Health Forum taxonomy [21].

### Analysis

At the review level, we categorized each systematic review by quality improvement category (Additional file 2). We analysed, summarised, and reported separately the results of all relevant comparisons within each systematic review using quantitative and qualitative methods as appropriate. We used vote counting as our method of analysis [22]. We counted the number of studies showing a positive direction of effect. If the review reported conflicting results or we were not able to determine the direction of effect, we classified the outcome as unclear. We excluded studies from our analysis that were beyond the scope of this project and reported this for each review.

We considered the results of the intervention effects featured in reviews when five or more of the included studies reported on a given outcome. We determined an intervention to be generally effective when the results of 67% or more of the included studies favoured the intervention; to have mixed effects when the results of 34% to 66% of the included studies favoured the intervention; or to be generally ineffective when the results of fewer than 34% of the included studies favoured the intervention. If there were fewer than five studies reporting on a specific intervention outcome within a systematic review, we considered this to be insufficient evidence to contribute to our overall analysis of that intervention.

## Results

### Results of the search

**Figure 1:**
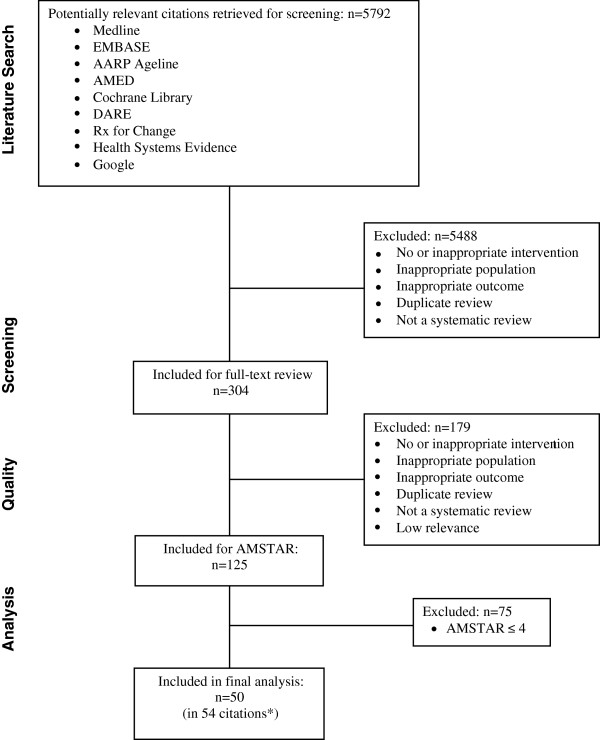
Flow chart of evidence from original source to final acceptance.

Figure 1 details the flow of information through the different stages of this overview using the ‘preferred reporting items for systematic reviews and meta-analyses’ (PRISMA) flow diagram [23]. Our searches resulted in 5,792 article citations. After the initial screening of titles and abstracts, we retrieved 304 articles for full-text review. Of these, we excluded 179 articles that did not meet the eligibility criteria. The remaining 125 systematic reviews were published in 76 journals between 1990 and 2011. We assessed the methodological quality of these systematic reviews and found a median AMSTAR score of 4 (interquartile range, 2 to 6). Fifty systematic reviews were reported in 54 papers (published in 27 different journals), had an AMSTAR score of 5 or more and were included in the overview, and are described in Additional file 3[16,17,24-75]. Details of the 75 excluded reviews are provided in Additional file 4.

### Description of systematic reviews included in analysis

Forty eight systematic reviews were published after 2000. All were published in English and originated from Australia [37], Belgium [71], Canada [16,43,54,62,64,67], China [49], Denmark [55], France [61], Germany [32], the Netherlands [63,69,70], Norway [36], Saudi Arabia [24], Switzerland [26], the United Kingdom [25,33,34,39,40,44-47,50-53,65],[66,68,72,73], and the United States [27,28,30,31,35,38,42,56-60],[74,75]. The number of included studies in each review ranged from five to eighty-two. Thirty-one reviews restricted study design to include only randomised controlled trials (RCTs) with or without controlled clinical trials (CCTs) [24-28,31,33,34,36-38,42,44,46],[47,51-53,55,56,58,60,61,64-67],[69,70,72,73]. In all but one review that included other study designs, the predominant study design adopted by the included primary studies was the RCT. All reviews except four [16,34,38,50] used more than one method of analysis and thirty-four reviews used meta-analyses [16,24-28,30,31,33,36-40,42,44-47],[49,54-56,59-62,64,67,69-73]. Patient education and support was the most common focus of the included reviews (n = 21), followed by telemedicine (n = 10), broad based reviews (n = 8), provider role changes (n = 7), and organisational changes (n = 4) (Table 1).

**Table 1:** Description of included reviews by intervention category

**Intervention category**	**No. of reviews included in analysis**	**Median AMSTAR score (min, max)**	**Range of publication year**	**Median no. of included studies within reviews (min, max)**
Patient education and support	21	8 (5, 11)	1990 to 2010	21 (5, 82)
Telemedicine	10	5.5 (5, 9)	2004 to 2011	20.5 (7, 44)
Provider role changes	7	7 (6, 9)	2003 to 2011	18 (5, 36)
Organisational changes	4	7.5 (6, 9)	1998 to 2011	7 (6, 23)
Broad based reviews	8	6 (5, 9)	2001 to 2011	41 (9, 58)

Twenty-six systematic reviews focused on type 1 or type 2 diabetes or both [24,27,28,30,31,33,38,39],[43-46,49,51,52,54,56,57,59],[61-64,68,70,74], five reviews considered patients with type 1 diabetes only [32,35,65,67,72], and 16 focused only on type 2 diabetes [16,25,26,36,37,42,47,50],[53,55,58,60,66,69,71,73]. Two systematic reviews did not specify the type of diabetes examined [40,75] and one review addressed a range of chronic conditions that included diabetes as an identifiable subset [31]. Interventions were directed to specific populations or a combination of patients (including family and carers), providers, and the healthcare system.

All but three reviews reported on glycaemic control in patients [33,69,75]. Twenty systematic reviews reported on changes in vascular risk factors (cholesterol, systolic and diastolic blood pressure) [16,30,33,36,37,43,45,47],[52-54,57-60,63,66,70,71,74]. Ten systematic reviews reported on optimal process of care goals [16,28,43,50,57,58,63,69],[71,75], with six reporting on HbA1c monitoring [16,28,50,57,63,71]. There was considerable heterogeneity among the included primary studies in terms of study designs, settings and interventions.

### Quality of systematic reviews included in the analysis

The majority of reviews met AMSTAR criterion relating to the following: conducting a comprehensive search of the literature (49/50); providing a list of the characteristics of included studies (49/50); using appropriate methods to combine the findings of the included studies (48/50); assessing and documenting the scientific quality of included studies (47/50), and using the results of the scientific quality assessment in formulating conclusions (38/50). However fewer studies met AMSTAR criteria relating to the following: using independent reviewers for selecting studies and extracting data (29/50); using publication status as part of the inclusion criteria (25/50); assessing the likelihood of publication bias (22/50); providing an a priori design (21/50), and providing a list of the included and excluded studies (18/50). Only one review met the criteria for reporting conflict of interest of review and studies (Table 2).

**Table 2:** AMSTAR score by QI category and individual systematic review

**Review**	AMSTAR question (Q)*											
	**Q1 A priori design provided**	**Q2 Duplicate study selection and data extraction**	**Q3 Comprehensive literature search**	**Q4 Publication status as inclusion criterion**	**Q5 List of studies (include and excluded) provided**	**Q6 Characteristics of the included studies provided**	**Q7 Quality assessment**	**Q8 Quality used appropriate**	**Q9 Methods used to combine appropriate**	**Q10 Publication bias assessed**	**Q11 Conflict of interest stated**	**Total**
**Patient education and support**												
Allemann 2009 [26]	y	y	y	n	y	y	y	y	y	y	n	9
Armour 2005 [27]	n	n	y	n	n	y	y	y	y	n	n	5
Brown 1990 [30]	n	y	y	y	n	y	y	n	y	n	n	6
Cooper 2009 [34]	n	n	y	y	y	y	y	y	y	n	n	7
Couch 2008 [35]	y	y	y	y	y	y	y	y	y	y	n	11
Deakin 2005 [36]	y	y	y	y	y	y	y	y	y	y	n	10
Duke 2009 [37]	y	y	y	y	y	y	y	y	y	n	n	9
Ellis 2004 [38]	n	y	y	y	n	y	n	n	y	y	n	6
Gary 2003 [42]	n	n	y	y	n	n	y	y	y	y	n	6
Hampson 2001 [45]	y	y	y	y	n	y	y	y	y	n	n	8
Harkness 2010 [46]	n	n	y	n	n	y	y	n	y	y	n	5
Hawthorne 2008 [47]	y	y	y	y	y	y	y	y	y	y	y	11
Loveman 2003 [52]	y	n	y	y	y	y	y	y	y	n	n	8
Loveman 2008 [53]	y	n	y	n	y	y	y	y	y	n	n	7
Minet 2010 [55]	n	n	y	n	n	y	y	n	y	y	n	5
Norris 2001 [58]	n	n	y	n	n	y	y	y	y	n	n	5
Norris 2002 [59]	y	y	y	n	y	y	y	y	y	n	n	8
Norris 2005 [60]	y	n	y	y	y	y	y	y	y	y	n	9
Savage 2010 [65]	n	n	y	y	n	y	y	n	y	n	n	5
Valk 2001 [69]	y	y	y	n	y	y	y	y	y	y	n	9
Winkley 2006 [72]	y	y	y	y	n	y	y	y	y	y	n	9
Total (n = 21)	12	11	21	13	11	20	20	16	21	11	1	
**Telemedicine**												
Balas 2004 [28]	n	n	y	y	n	y	y	y	n	n	n	5
Farmer 2005 [39]	n	n	y	n	n	y	y	y	y	n	n	5
Liang 2011 [49]	n	n	y	n	n	y	y	y	y	n	n	5
Montori 2004 [56]	y	y	y	y	n	y	n	n	y	n	n	6
Polisena 2009 [62]	y	y	y	y	n	y	y	y	y	n	n	8
Russell 2009 [64]	n	n	y	y	n	y	y	y	n	n	n	5
Shulman 2010 [67]	n	y	y	y	y	y	y	y	y	n	n	8
Sutcliffe 2011 [68]	y	n	y	y	n	y	y	y	y	n	n	7
Verhoeven 2007 [70]	n	y	y	n	n	y	y	n	y	n	n	5
Wu 2010 [73]	y	n	y	n	y	y	y	n	y	y	n	7
Total (n = 10)	4	4	10	6	2	10	9	7	8	1	0	
**Provider role changes**												
Alam 2009 [25]	y	y	n	n	n	y	y	y	y	y	n	7
Clark 2011 [33]	n	y	y	n	n	y	y	y	y	y	n	7
Lindenmeyer 2006 [50]	n	y	y	y	n	y	y	y	y	n	n	7
Loveman 2003 [51]	y	y	y	y	y	y	y	y	y	n	n	9
Machado 2007 [54]	n	y	y	n	n	y	y	y	y	y	n	7
Norris 2006 [57]	y	n	y	n	n	y	y	y	y	n	n	6
Wubben 2008 [74]	n	y	y	n	n	y	y	y	y	n	n	6
Total (n = 7)	3	6	6	2	1	7	7	7	7	3	0	
**Organisational**												
Al-Ansary 2011 [24]	n	y	y	y	y	y	y	n	y	n	n	7
Clar 2007 [32]	y	y	y	n	y	y	y	y	y	y	n	9
Foy 2010 [40]	n	y	y	n	n	y	y	y	y	y	n	7
Griffin 1998 [44]	n	n	y	y	n	y	y	y	y	n	n	6
Total (n =4)	1	3	4	2	2	4	4	3	4	2	0	
**Broad based reviews**												
Chodosh 2005 [31]	n	y	y	n	n	y	y	y	y	y	n	7
Glazier 2006 [43]	n	y	y	n	n	y	y	n	y	n	n	5
Pimouguet 2011 [61]	n	n	y	n	y	y	y	y	y	y	n	7
Renders 2001 [63]	n	n	y	n	n	y	y	y	y	n	n	5
Saxena 2007 [66]	n	y	y	y	n	y	y	n	y	n	n	6
Shojania 2006 [16]	n	y	y	n	n	y	n	n	y	y	n	5
Vermeire 2005 [71]	n	y	y	y	y	y	y	y	y	y	n	9
Zhang 2007 [75]	y	n	y	n	n	y	y	y	y	y	n	7
Total (n = 8)	1	5	8	2	2	8	8	5	8	5	0	

* We awarded one point to each item that scored ‘yes’ (y) and summed these to calculate a total score; n, no.

A summary of the outcome results from each review is provided in Table 3. Detailed descriptions of the findings for each review are provided in Additional file 3.

### Synthesis of broad based reviews

We identified eight systematic reviews evaluating the effectiveness of a range of QI interventions directed to one or more of patients, providers, and healthcare systems (Additional file 3) [16,31,43,61,63,66,71,75]. Three reviews only included RCTs with or without CCTs. Patient-directed interventions involved education or information provision; [16,31,43,61,63,71], provider-targeted interventions included the use of reminders or prompts, educational materials, meetings, and outreach [16,43,63,66,75]; system-targeted interventions included changes in the physical structure of healthcare facilities, introduction of health records systems or registries, and changes to the site of service delivery [16,63,75]. Three reviews examined the effectiveness of multiple approaches in specific patient populations: older adults [31], socially disadvantaged groups [43], and minority ethnic groups [66]. Among these reviews, we noted that provider role changes such as role expansion and use of multidisciplinary teams [16,31,43,61,63,66,71,75], and telemedicine [16,43,63,75] interventions were frequently evaluated. These reviews observed a number of likely effective interventions. For example, Shojania and colleagues [16,17] reviewed 66 studies (including 50 RCTs) and 11 different QI interventions and observed a mean reduction in HbA1c of 0.42%. Meta-regression identified two interventions that were associated with HbA1c reductions greater than 0.5%, team changes, and case management.

### Synthesis of results by intervention category

#### Patient education and support

Twenty-one systematic reviews examined the impact of patient education and support interventions that help patients and their families or carers understand diabetes and its treatment by providing education and information, emotional and behavioural support, coping strategies, and self-management training [26,27,30,34-38,42,45-47,52,53],[55,58-60,65,69,72] (Additional file 3). The majority of reviews only included RCTs with or without CCTs (n = 17). Reviews varied in terms of the content and variety of interventions, the target population, the provider of the intervention, and the setting. We identified reviews evaluating tailored education packages including culturally appropriate education [47], education for adolescent diabetes patients [45], support for children vs adults [72], individual vs group education [36,37,42], and education delivered in the community, home, recreational camps, and worksite [59]. The deliverers of patient education interventions, when specified, were nurses (24.8%), dieticians (21.1%), physicians (16.6%), psychologists (12.5%), multidisciplinary teams (8.0%), diabetes educators (7.4%), specialist nurses (3.7%), or others (5.9%). Four reviews did not specify who delivered the intervention [26,27,34,65]. Changes in HbA1c levels in patients were reported in all reviews, with one exception [69]. Ten reviews reported on vascular risk factor control in patients [30,36,37,45,47,52,53,58-60] and one review focused on diabetic foot outcomes [69].

Overall, patient education and support interventions were associated with improved glycaemic control for patients of all ages in 18 reviews [26,27,30,35-38,42,45-47,52,53],[55,58-60,65,72], and with mixed results in two reviews [60,72] (Table 3). Improved blood pressure [36,52,53] and cholesterol [30,47,52,53,58] levels were also associated with this intervention, though we noted mixed results for blood pressure in one review [37] and cholesterol in another [51]. A decrease in the occurrence of diabetic foot outcomes such as ulcerations, infections, and amputations was also associated with patient education and support [69].

**Table 3:** Summary of results from included reviews on outcomes

**Review**	**Quality**^**a**^	**Glycaemic control**		**Vascular risk factor control**		**Retinopathy screening**	**Foot screening**	**Renal monitoring**	**Smoking**	
		**Process**	**Patient**	**Process**	**Patient**				**Process**	**Patient**
**Patient education and support**										
Allemann 2009 [26]	9		+							
Armour 2005 [27]	5		+							
Brown 1990 [30]	6		+		+ (Lipids), 0 (BP)					
Cooper 2009 [34]	7		0							
Couch 2008 [35]	10		+							
Deakin 2005 [36]	10		+		+ (BP), 0 (Lipids)					
Duke 2009 [37]	9		+		Mixed (BP), O (Lipids)					0
Ellis 2004 [38]	6		+							
Gary 2003 [42]	6		+							
Hampson 2001 [45]	8		+		0 (Lipids)					
Harkness 2010 [46]	5		+							
Hawthorne 2008 [47]	11		+		+ (Lipids) 0 (BP)					
Loveman 2008 [53]	7		+		+ (Lipids, BP)					
Loveman 2003 [52]	8		+		Mixed (Lipids) + (BP)					
Minet 2010 [55]	5		+							
Norris 2005 [60]	9		Mixed		0 (Lipids, BP)					
Norris 2002 [59]	8		+		0 (Lipids, BP)					
Norris 2001 [58]	5		+		+ (Lipids) 0 (BP)		0			
Savage 2010 [65]	5		+							
Valk 2001 [69]	9						*			
Winkley 2006 [72]	9		Mixed							
**Telemedicine**										
Balas 2004 [28]	5	0	+			0	0			
Farmer 2005 [39]	5		Mixed							
Liang 2011 [49]	5		+							
Montori 2004 [56]	6		+							
Polisena 2009 [62]	8		Mixed							
Russell 2009 [64]	5		+							
Shulman 2010 [67]	8		+							
Sutcliffe 2011 [68]	7		+							
Verhoeven 2007 [70]	5		Mixed		0 (Lipids, BP)					
Wu 2010 [73]	7		+							
**Provider role changes**										
Alam 2009 [25]	7		+							
Clark 2011 [33]	7			Unclear	+ (BP)					
Lindenmeyer										
2006 [50]	7	0	+			0	0			
Loveman 2003 [51]	9		+							
Machado 2007 [54]	7		+		+ (Lipids, BP)					
Norris 2006 [57]	6	0	+		0 (Lipids, BP)					
Wubben 2008 [74]	6		+		+ (Lipids, BP)					
**Organisational changes**										
Al-Ansary 2011 [24]	7		0							
Clar 2007 [32]	9		0							
Foy 2010 [40]	7		+							
Griffin 1998 [44]	6		0							
**Broad based reviews**										
Chodosh 2005 [31]	6		+							
Glazier 2006 [43]	5		+		0 (Lipids, BP)	0	0	0		
Pimouguet 2011 [61]	7		+							
Renders 2001 [63]	5	+	+	0	0 (Lipids, BP)	+	0	0		
Shojania 2006 [16]	5	0	+		0 BP	0	0			
Saxena 2007 [66]	6		+		+ (Lipids) mixed (BP)					
Vermeire 2005 [71]	9		0		0 (Lipids, BP)					0
Zhang 2007 [75]	7					+				

^**a**^AMSTAR score; **+,** generally effective based on n ≥ 5 comparisons; 0, insufficient comparisons to draw conclusions about effectiveness. *The Valk review reported favourable patient outcomes that may reflect screening activities. See Additional file 3 for further details of this review. *BP* blood pressure.

#### Telemedicine

We identified ten systematic reviews examining the effectiveness of telemedicine technology in the provision of diabetes care to local and remotely based patients (Additional file 3) [28,39,49,56,62,64,67,68],[70,73]. These interventions consisted of the transmission of blood glucose values by patients via phone (including mobile or fax) and computer (Internet or website) to healthcare providers for review, with feedback to patients by phone, videoconference, or other electronic means. Seven reviews only included RCTs with or without CCTs. Two reviews addressed interventions targeted only to children and young adults [67,68]. Three reviews described a system interface where data were transmitted to a remote server for analysis, after which appropriate automated messages or reminders were sent to patients or their providers [28,39,70].

All reviews reported on the clinical effectiveness of the interventions on glycaemic control in patients. Telemedicine interventions improved HbA1c levels in eight reviews [28,49,56,64,67,68,70,73], and three reviews had mixed results [39,62,70]. Where reviews examined different modes of data transmission, it was found that SMS (short message systems), when used alone or in conjunction with the Internet to deliver home glucose records and support, were generally associated with improved glycaemic control in patients [49,68]. Internet as a primary means of transmission of blood glucose data and support also had a positive effect on glycaemic control [68].

#### Provider role changes

Seven systematic reviews examined the effectiveness of changing, expanding, or integrating the roles of healthcare professionals to improve diabetes care and outcomes (Additional file 3) [25,33,50,51,54,57,74]. Three reviews only considered RCTs with or without CCTs. Five systematic reviews examined the impact of role expansion by increasing the responsibilities of pharmacists to include medication management, patient education, and support [50,54,74] and expanding the role of the nurse to include educating and monitoring diabetes patients [33,51]. Other provider role change interventions included role substitution and the use of multidisciplinary healthcare teams. In one of these reviews, local community health workers substituted for medical professionals or worked as part of a multidisciplinary team to provide socioeconomic or culturally appropriate care [57]. In a second review, general practitioners substituted for psychological specialists [25].

All reviews reported on the clinical effectiveness of the interventions on glycaemic control or vascular risk factor control in patients. Interventions that involved changing, expanding, or integrating the roles of healthcare professionals were associated with improvements in patients’ glycaemic [25,50,51,54,57,74], cholesterol [54,74], and blood pressure levels [33,54,74].

#### Organisational changes

Four systematic reviews examined the effectiveness of organisational changes to improve diabetes management and patient care (Additional file 3) [24,32,40,44]. Two reviews only considered RCTs with or without CCTs. Reviews compared the effectiveness of hospital vs home-based [32] specialist or general practice care [44], point of care testing for HbA1c at the time of patient consultation [24], or shared decision making between primary care physicians and specialists in patients receiving ambulatory care [40]. The interventions in these systematic reviews were heterogeneous and included the use of patient/provider reminder systems, protocols, multidisciplinary teams, and interactive communication between primary care physicians and specialists. One review focused solely on outpatient paediatric populations [32]. Changes to patient HbA1c levels were the only outcome reported in these systematic reviews.

The systematic reviews in this intervention category had relatively few included studies and thus provided insufficient evidence to allow us to determine if the organisational changes were associated with improved glycaemic control in patients. Only one systematic review included sufficient studies showing that shared decision making between primary care physicians and specialists improved blood glucose levels [40].

## Discussion

### Summary of the evidence

In this overview, we identified 125 systematic reviews that evaluated the effectiveness of quality improvement interventions to improve diabetes care. We excluded 75 of these from further assessment due to low methodological quality and undertook a detailed analysis of 50 high quality reviews. The majority of included reviews only considered RCTs with or without CCTs; in the remaining reviews that included other designs, RCTs and CCTs were the commonest designs included.

The eight broad based reviews examined a range of different interventions that led to improvements in patient self-management outcomes (glycaemic control and cholesterol levels) and process of care behaviours (HbA1c and retinopathy monitoring) with mixed results for blood pressure control. Based on our assessment of 42 high quality intervention specific reviews, patient education and support interventions were shown to improve HbA1c, blood pressure, cholesterol, and diabetic foot outcomes in patients; telemedicine interventions were associated with improved glycaemic control in patients, and provider role change interventions improved glycaemic and vascular risk factor control in patients. It was unclear what impact organisational interventions had on glycaemic control in patients. It was also unclear if there was a relationship between the above four interventions and improvements in monitoring of HbA1c, vascular risk factors, or retinopathy, or diabetic foot outcomes. The majority of reviews only included randomised trials alone (with or without controlled clinical trials). In general, the results and conclusions of the systematic reviews that only included RCTs with or without CCTs were similar to those including a broader range of designs. We were unable to identify any high quality reviews that focussed on other QI interventions relevant to our objectives, such as financial or regulatory interventions.

### Strengths and weaknesses of the overview

Our objective was to synthesise a comprehensive body of published evidence in a single overview. This is a challenging undertaking considering that overview methods are still evolving and a variety of approaches are being used [20,76,77]. Strengths of our approach include the use of explicit methods to identify, appraise and summarise available systematic reviews of interventions to improve diabetes management and outcomes. We employed sensitive search strategies that were developed and run by an information specialist with expertise in searching for interventions to improve health care delivery and healthcare systems. Two authors independently undertook study selection, quality appraisal using the validated AMSTAR tool, and data abstraction with a consensus process to address disagreements. Further, we performed a reanalysis of the results of reviews using vote-counting to ensure consistency of analytical approach when considering results across the reviews. We focussed on high quality reviews (any review scoring ≥5 on AMSTAR) as our previous experience suggested that reviews with lower AMSTAR scores are difficult to interpret and likely do not provide reliable evidence. Our approach allowed us to explore whether different review teams addressing similar review questions independently observe similar results and draw broadly similar conclusions. The consistency of findings and conclusions across high quality reviews, for example that patient education and support improves many aspects of diabetes care, suggests that this is not likely a spurious finding due to the review team or methods chosen.

However, overviews inevitably suffer from potential weaknesses, some of which are common to all synthesis projects and others that are specific to overviews. Potential weaknesses that are common to all syntheses include the possibility that our searches missed relevant reviews, or that errors were made during study selection, quality appraisal and data collection. Overview-specific weaknesses relate to the fact that our unit of study is a completed systematic review. Thus, overview authors are dependent upon the methods of the included systematic reviews that potentially suffer from some of the common weaknesses mentioned above. Overviews that consider multiple reviews on the same topic potentially protect against weaknesses in individual reviews unless all included reviews share precisely the same methodological weakness. In our overview we also tried to minimise the likelihood that we would be misled by individual reviews by excluding low quality reviews that are more likely to suffer to from major methodological weaknesses.

Overviews are also dependent on the reporting of the included systematic reviews, which limits the granularity of detail available to the overview author. As a result, the overview author needs to trust the systematic review authors' quality appraisal and data abstraction and is limited by the level of detail in the original systematic reviews when describing characteristics of included studies such as setting, and intervention, etcetera. As well, overviews are limited by the coverage of the identified systematic reviews. For instance, there has been increasing interest in the use of financial interventions to improve diabetes care; the United Kingdom introduced a pay-for-performance incentive program in 2004, rewarding family practices for achieving performance targets in chronic disease management, including diabetes [78]. However, despite this policy interest and the availability of primary studies, we were unable to find any high quality systematic reviews that addressed the effects of financial interventions on quality of diabetes care. Finally, overview authors should expect considerable overlap in the primary studies summarised in the included systematic reviews. As a result it is important not to treat systematic reviews as independent observations but rather see included systematic reviews as a different lens addressing the same question to determine whether different teams draw broadly similar conclusions.

The interventions examined in these reviews were frequently complex. Reporting of complex interventions is often poor in primary studies [79] and even poorer in systematic reviews that may reduce a short description in a primary study to a few words in a table. This creates a number of difficulties for overview authors; whilst overview authors might be confident that a review does address the overview question frequently, there will be insufficient detail within the review to be able to describe the evaluated reviews in detail, or to determine effective components of complex interventions, or explore potential effect modifiers.

Within our overview, we reanalysed the included reviews using vote counting based on direction of effect. The weaknesses of vote counting are well documented and include the failure to provide an estimate of the effect size of an intervention, failure to take into account the precision of the estimate from the primary comparisons, and giving equal weight to comparisons with 100 or 1,000 participants [22]. Nevertheless, we would argue that vote counting provided a flexible approach to consider effectiveness across reviews with few assumptions given that the included reviews involved different study designs, presented individual study reviews using different metrics and used a variety of analytical approaches (including descriptive analysis, vote-counting, meta-analysis and meta-regression). To increase the confidence, we reported separately, the number of RCTs that contributed to the overall effect for each intervention comparison. Further, we also reported the results of any meta-analyses performed in the reviews that had bearing on the interventions and outcomes of interest.

### Implications for policy

Overviews are high-level syntheses of research evidence that provide an evidence map for decision makers and high-level conclusions about an issue. However, it is likely that decision makers will also need to consult some of the included systematic reviews, and potentially the individual studies, to address their specific questions. Thus, this synthesis is best seen as an entry point to evidence to inform healthcare decision makers' policy options about interventions to improve diabetes outcomes. Current initiatives to improve diabetes management and care should be informed by this evidence base. There is consistent evidence that a number of interventions, for example, patient education and support, telemedicine, and provider role changes, appear to improve diabetes quality of care, and policy-makers need to consider whether their current services optimally provide these effective interventions to their population of persons with diabetes. Overall, there is some evidence to suggest that tailoring interventions to specific cultural or age groups may be beneficial to the effectiveness of the treatment strategy. Interventions at an organisational level had insufficient evidence from which to draw conclusions about their effectiveness, and therefore policy makers should be cautious about implementing these approaches until further evidence is available.

### Implications for research

There is a considerable body of evidence evaluating interventions to improve diabetes care that should inform future research. Researchers conducting both primary studies and systematic reviews of diabetes quality improvement interventions should consider the breadth of outcomes relevant to excellent diabetes care. In areas with a substantial number of trials demonstrating benefit, for instance, patient education and support, future research should focus on direct comparisons of different delivery methods for the same intervention or of direct comparisons of the relative effectiveness of different interventions.

The results of available systematic reviews should inform the choice and design of evaluated interventions and evaluative methods. For example, new systematic reviews of diabetes QI interventions might focus on interventions where there are few or no current systematic reviews. Patient education provides an example of a group of interventions where the depth and quality of the evidence is abundant and where further reviews may add little to our knowledge unless they address secondary questions such as how to optimise or extend the reach of patient education and support interventions. While we identified some evidence of attempts by researchers to separate the effects of complex interventions, future studies should further focus on such separation in order to provide specific advice on how to optimise efforts to improve diabetes management. Given our observation that 60% of the reviews assessed for this project were rejected based on low AMSTAR scores, researchers are strongly encouraged to consider AMSTAR assessment criteria during the systematic reviewing process in order to improve the methodological quality and/or reporting of their work.

## Conclusions

Overviews provide high-level summaries of empirical research. This overview identified and summarised the best current available evidence from 125 systematic reviews on the effectiveness of different QI interventions to improve diabetes care. The results suggest that patient education and support, provider role changes and telemedicine are associated with improved patient outcomes. They can be used by decision makers to identify policy options to improve diabetes care and as a source document to identify systematic reviews and individual studies that are relevant to their context and that address their specific questions. It also identified potential areas for future research; highlighting the problem of (likely) inappropriate duplication of effort between existing systematic reviews and the lack of high-quality systematic reviews addressing interventions of policy interest, such as regulatory or financial interventions.

## Abbreviations

AMED: Allied and Complementary Medicine; AMSTAR: Assessment of multiple systematic reviews; CCT: Controlled clinical trials; DARE: Database of Abstracts and Reviews; EPOC: Effective Practice and Organisation of Care; HbA1c: Glycated haemoglobin; PRISMA: Preferred reporting items for systematic reviews and meta-analyse; QI: Quality improvement; RCT: Randomised controlled trial; SMS: Short message system.

## Competing interests

The authors declare that they have no competing interests. JMG is an author on one of the included reviews.

## Authors’ contributions

JMG, AM and MCW conceptualised the project. MF developed and executed the search strategy. JW, SCW, RB, AM, MCW and JMG screened articles for potential inclusion. JW, SCW, RB, AM and MCW appraised the quality of articles. JW, SCW, RB, AM, KJS and MCW extracted data from the included reviews. JMG, JW, SCW, KJS and MF drafted and revised the manuscript. RB, JW, KJS prepared the tables and figures. All authors had full access to all data, contributed to revision of the manuscript and approved the final version. JMG is the guarantor. All members of the Diabetes QI Overview Team provided additional support and/or carried out specific project-related tasks. All authors read and approved the final manuscript.

## Authors’ information

JMG holds a Canada Research Chair in Health Knowledge Transfer and Uptake.

## Supplementary Material

Additional file 1 **Search strategies.** The full search strategies used in the evidence search. Click here for file

Additional file 2 **Intervention categories identified.** Description of the intervention categories used to classify reviews. Click here for file

Additional file 3 **Details of included reviews.** Characteristics and results of the included reviews. Click here for file

Additional file 4 **List of excluded reviews.** Bibliography of excluded reviews (reviews that scored less than 5 using AMSTAR tool). Click here for file
