# Supplementary material for: Improving quality of care for persons with diabetes: an overview of systematic reviews - what does the evidence tell us?
Source: Syst Rev. 2013 May 7;2:26. doi: 10.1186/2046-4053-2-26 (PMC3667096; doi:10.1186/2046-4053-2-26)
Supplement: Additional file 1 — Search strategies. The full search strategies used in the evidence search. [file 2046-4053-2-26-S1.pdf]

Additional file 1 - Search strategies

We used two strategies, Strategy 1 and Strategy 2, to identify systematic reviews and applied a methodological filter for systematic reviews to both strategies. Search dates spanned 1976 to April 2011.

Strategy 1 (Appendix 1A) is based on the PubMed strategy published in a report by Shojania et al. [16, 17] Strategy 1 uses the *diabetes* and *quality improvement* terms, from the Shojania strategy, but omits the following parts of the strategy: title search for *sugar*, *glycemia*, and *glycemic*; the methodological filter for trials; a search of journal titles; and a search of authors. Strategy 1 intermingles controlled vocabularies from both ML (Medical Subject Headings--MeSH) and EM (EMTREE)—e.g. MeSH terms were searched in EMBASE and EMTREE terms were searched in Medline. This search covers the period from 1976 to June 2009.

Strategy 2 (Appendix 1B) uses terms not used in the Shojania study to identify additional quality improvement interventions. This strategy is based on search strategies and methodologies developed within the Cochrane Effective Practice and Organisation of Care (EPOC) Group. The Medline version of Strategy 2 has been translated for and run in the databases listed above and controlled vocabulary terms customised for each database. Strategy 2 identifies approximately 35% of the reviews screened in full text; and 36% of the reviews included in this overview. This search covers the period from 1976 to April 2011.

Appendix 1: A – Search Strategy 1

Search date: June 23, 2009

Databases: HealthSTAR (OVID), EMBASE, Ovid MEDLINE(R) Daily Update, Ovid MEDLINE(R)

Abbreviations: ML=Medline term (MeSH); EM=EMBASE term (EMTREE)

Limits: 1976-

Note: MeSH are used in the HealthStar database.

1 exp *diabetes mellitus/ or exp *diabetic angiopathy/ or exp *pregnancy diabetes mellitus/ [EM] (448363)

2 diabetes mellitus/ or exp diabetes mellitus, type 1/ or exp diabetes mellitus, type 2/ or diabetes, gestational/ or diabetic ketoacidosis/ or prediabetic state/ [ML] (515988)

3 (diabet$ or prediabet$).ti,ab. (685104)

4 or/1-3 (793844)

5 exp Disease Management/ or exp Patient-Centered Care/ or exp Primary Health Care/ or exp Progressive Patient Care/ or exp Critical Pathways/ or exp Delivery of Health Care, Integrated/ or exp Health Services Accessibility/ or exp Managed Care Programs/ or exp Product Line Management/ or exp Patient Care Team/ or exp Behavior Control/ or exp Counseling/ or exp Health Promotion/ or exp Patient Compliance/ or exp After-Hours Care/ [ML] (2228827)

6 exp *disease management/ or exp *patient care/ or exp *Primary Health Care/ or Progressive patient care/ or exp *Clinical Pathway/ or exp *Health Care Delivery/ or exp *Managed Care/ or exp *Behavior Control/ or exp *Counseling/ or exp *Health Promotion/ or Patient Compliance/ [EM] (1417731)

7 (coordination or coordinated or Multifactorial or Multi-factorial or Multicomponent or Multi-component or multidisciplinary or multi-disciplinary or interdisciplinary or inter-disciplinary or integrated or community-based or organized).ti. (106975)

8 (care or approach or intervention or strategy or strategies or management or managing or center* or clinic*).ti. or "Organization and Administration"/ [ML] (2858930)

9 7 and 8 (28335)

10 5 or 6 or 9 (2981646)

11 exp Total Quality Management/ or exp Quality control/ [ML EM] (214114)

12 (quality and (continuous or total) and (management or improvement)).ti. (2242)

13 (tqm or cqi).ti. (1078)

14 or/11-13 (215278)

15 exp Education, Continuing/[ML] (101551)

16 exp continuing education/ [EM] (101551)

17 (Education and Continuing and (medical or professional* or nursing or physician* or nurse*)).ti. (7983)

18 (outreach and (visit* or educational)).ti. (204)

19 (academic and detailing).ti. (180)

20 or/15-19 (102957)

21 exp Diffusion of Innovation/ [ML] (145812)

22 (diffusion and (innovation or technology)).ti. (388)

23 or/21-22 (145973)

24 exp Medical audit/ [ML EM] (36413)

25 ((Audit or feedback or compliance or adherence or training) and (improvement$ or improving or improves or improve or guideline$ or practice$ or medical or provider$ or physician$ or nurse$ or clinician$ or academic or visit$)).ti. or exp practice guidelines/ (277211)

26 ((financial or economic or physician$ or patient$) and incentive$).ti. or exp Reimbursement Mechanisms/ (63203)

27 or/24-26 (367481)

28 exp Medical informatics/ [ML EM] (374410)

29 (computer or (decision and support)).ti. (92489)

30 exp Telemedicine/ or Telemedicine.ti. or telecommunication$.ti. or exp Internet/ or web.ti. or modem.ti. or telephone$.ti. or exp telephone/ [ML EM] (141973)

31 or/28-30 (558712)

32 or/10,14,20,23,27,31 (3872555)

33 meta-analysis.pt. (41545)

34 meta-analysis/ or systematic review/ or meta-analysis as topic/ or exp technology assessment, biomedical/ (110729)

35 ((systematic* adj3 (review* or overview*)) or (methodologic* adj3 (review* or overview*))).ti,ab. (61977)

36 ((quantitative adj3 (review* or overview* or synthes*)) or (research adj3 (integrati* or overview*))).ti,ab. (8445)

37 ((integrative adj3 (review* or overview*)) or (collaborative adj3 (review* or overview*)) or (pool* adj3 analy*)).ti,ab. (13512)

38 (data synthes* or data extraction* or data abstraction*).ti,ab. (21096)

39 (mantel haenszel or peto or der simonian or dersimonian or fixed effect* or latin square*).ti,ab. (17638)

40 (met analy* or metanaly* or health technology assessment* or HTA or HTAs).ti,ab. (3686)

41 (meta regression* or metaregression* or mega regression*).ti,ab. (2187)

42 (meta-analy* or metaanaly* or systematic review* or biomedical technology assessment* or bio-medical technology assessment*).mp,hw. (164293)

43 (medline or cochrane or pubmed or medlars).ti,ab,hw,jn. (110716)

44 (cochrane or health technology assessment or evidence report).jw. (14024)

45 (evidence adj3 (review? or appraisal? or overview* or synthesis)).ti,ab. (37543)

46 (critical adj3 (review? or appraisal? or overview?)).ti,ab. (32159)

47 ((synthes$ or evaluat$) adj2 evidence$).ti,ab. (9030)

48 or/33-47 (348481)

49 4 and 32 and 48 (4675)

50 limit 49 to yr="1976-current" (4672)

51 limit 50 to human (4547)

52 limit 51 to humans (4547)

53 remove duplicates from 52 (3668)

54 from 53 keep 1 (1)

55 from 53 keep 2-662 (661)

56 from 53 keep 663-3668 (3006)

Appendix 1: B - Strategy 2 (Medline and Embase)

Medline

Search Date: April 2011

Database: OVID Medline(r) In-Process & Other Non-indexed Citations and OVID Medline(R) <1948 to present>

Limits: 1976-

1 exp diabetes mellitus/ or diabet$.ti. (279488)

2 intervention?.ti. (53099)

3 (intervention? adj6 (clinician? or collaborat$ or community or complex or doctor? or educational or family doctor? or family physician? or family practitioner? or financial or gp or general practice? or hospital? or impact? or improv$ or individuali?e? or individuali?ing or interdisciplin$ or multicomponent or multi-component or multidisciplin$ or multi-disciplin$ or multifacet$ or multi-facet$ or multimodal$ or multi-modal$ or personali?e? or personali?ing or pharmacies or pharmacist? or pharmacy or physician? or practitioner? or prescrib$ or prescription? or primary care or professional$ or provider? or regulatory or regulatory or tailor$ or target$ or team$ or usual care)).ab. (62544)

4 (multiintervention? or multi-intervention? or postintervention? or post-intervention? or preintervention? or pre-intervention?).ti,ab. (57835) not in ml 04-26-2011

5 exp drug therapy/ and irrational.ti,ab. [ml] (175)

6 (rational adj4 (drug therapy or "drug use" or prescribing)).ti,ab. (670)

7 (rational or irrational).ti. and drug therapy.hw. (347)

8 ((promote or prefer) adj5 generic).ti,ab. (40)

9 prescribing habits.ti,ab. (545)

10 ((chang$ or improv$ or optimi$ or optimal) adj3 prescrib$).ti,ab. (1927)

11 ((physician? or doctor? or nurse?) adj4 compliance).ti,ab. (1208)

12 (promoting.ti. and (health$ or care or education or nurse? or nursing or patient? or hospital$).ti,hw.) or (promoting and (doctor? or physician? or pharmacist?)).ti. (4638)

13 (fund-hold$ or fundhold$ or capitation or capitated or copay$ or co-pay$).ti,ab. (4359)

14 (impact or effectiveness or change? or changing).ti. (448106)

15 ((change? or changing or improv$ or effect$ or enhanc$ or influenc$) adj2 ((administrat$ or care or management or outcome or organi?ation$ or policy or policies or provider? or physician? or nurse? or nursing or pathway? or protocol?) adj5 (diagnos$ or healthcare or "health care" or hospital? or patient? or practice? or provider? or treatment?))).ti,ab. (47434)

16 (practice pattern? or ((change? or changing) adj2 practice)).ti,ab. (7589)

17 ((effect? or effectiveness or chang$ or improv$ or impact) adj3 practice).ti,ab. (16156)

18 (communit$ or team$ or interdisciplinar$ or multidisciplinar$).ti,ab. (346079)

19 (implementation or implementing).ti. and (care or healthcare).ti,hw. (5356)

20 (improv$ adj3 (diagnosis or treatment? or prescribing)).ti,ab. (45180)

21 (((evidence or evidence-based) adj4 intervention) or evidence-driven).ti,ab. (1548)

22 "practice-based".ti,ab. (2617)

23 (improv$ adj3 (decision$ or implement$ or health care or healthcare or initiative? or management or multifacet$ or multi-facet$ or multi-component or practi?e? or practitioner? or prescrib$ or prescription? or professional? or program? or programme? or provider?)).ti. (6436)

24 (improv$ adj2 (patient-care or family practice or ((family or general) adj2 (practi?e or practitioner? or doctor?)) or primary care)).ab. (3678)

25 recommended practice?.ti,ab. (592)

26 ((information or evidence) adj2 uptake).ti,ab. (333)

27 ((knowledge adj2 (application or broke$ or creation or diffus$ or disseminat$ or exchang$ or implement$ or management or mobili$ or translat$ or transfer$ or uptake or utili$)) or (evidence$ adj2 (exchang$ or translat$ or transfer$))).ti,ab. (6069)

28 (kt adj2 (application or broke$ or diffus$ or disseminat$ or decision$ or exchang$ or implement$ or intervent$ or mobili$ or plan$ or policy or policies or strateg$ or translat$ or transfer$ or uptake or utili$)).ti,ab. (126)

29 ((computer-tailored or individuali?ing or individuali?ed or personali?e? or personali?ing or tailor$) adj2 (feedback or intervention? or information or plan?)).ti,ab. (3601)

30 ((conventional or evidence-based or pattern or regular or routine or standard or traditional or usual) adj2 (care or healthcare or patient care or practice)).ti,ab. (43545)

31 (collaborative? or interdisciplin$ or inter-disciplin$ or multidisciplin$ or multi-disciplin$ or team? or team-based or skill-mix).ti. (31652)

32 ((collaborative or multidisciplinary or interdisciplinary) adj2 (care or healthcare or patient care or team?)).ab. (10849)

33 (skill? adj2 (mix or mixes)).ti,ab. (502)

34 (doctor-driven or doctor-led or gp-led or nurse-led or nurse-driven or pharmacist-led or pharmacist-driven or physician-led or physician-driven).ti,ab. (1755)

35 physician directed.ti,ab. (228)

36 (booklet? or leaflet$ or pamphlet$ or "written information").ti. or ((booklet? or leaflet$ or pamphlet$ or "written information") adj5 (intervention? or care or healthcare or physician? or practitioner? or provider?)).ab. (3068)

37 (academic detailing or e-detailing or (opinion? adj2 leader?)).ti,ab. (870)

38 ("audit and feedback" or ((physician? or doctor? or practitioner? or nurse? or provider?) adj feedback)).ti,ab. (453)

39 reminder?.ti. (1076)

40 (reminder? adj2 (clinician? or physician? or practitioner? or nurse? or doctor? or provider?)).ab. (255)

41 ((clinician? or physician?) adj2 (prompt or prompts or prompting)).ti,ab. (417)

42 ((doctor? or nurse? or pharmacist? or physician? or practitioner?) adj2 behavio?r?).ti,ab. (2393)

43 (nurse? adj4 substitut$).ti,ab. (97)

44 physician's practice patterns/ (32275)

45 (nurse-practitioner? or physician? assistant?).ti. (4396)

46 ((doctor? or nurse? or pharmacist? or physician?) adj2 role?).ab. (4656)

47 ((nurse? or physician? or pharmacist? or provider?) adj2 initiative?).ti,ab. (270)

48 (virtual reality or vr training or vr simulat$ or (simulat$ adj2 skill?)).ti,ab. (3261)

49 (blog$ or wiki$ or pda or "palm pilot?" or podcast$ or blackberr$ or twitter or tweet or tweeting or facebook or social networking or social marketing or youtube).ti,ab. or blogging/ (6727)

50 (health 20 or healthcare 20 or health care 20 or web 20).ti,ab. (250)

51 (((individuali$ or integrated) adj2 (care or healthcare or medical care)) or patient-centred or patient-centered or patient-control$).ti,ab. (13300)

52 quality improvement.ti,ab. (10764)

53 *patient satisfaction/ (15924)

54 patient education as topic/ (61135)

55 (algorithm? and (care or healthcare or patient?)).ti,hw. (10728)

56 education, pharmacy, continuing/ or education, medical, continuing/ or education, nursing, continuing/ or education, professional, continuing/ (38284)

57 (continuing adj2 education adj3 (physician? or nurse? or nursing or practitioner? or doctor? or family physician? or general practitioner? or family doctor? or primary care or primary healthcare)).ab. or (continuing adj3 education).ti. (8287)

58 (((continuing or "on the job" or "off the job" or postgrad$ or post-grad$ or resident? or intern? or internship? or workplace) adj2 (education$ or training)) or (skill? adj (education or training))).ti,ab. (24800)

59 (reminder? adj2 (clinician? or physician? or practitioner? or nurse? or doctor? or provider?)).ab. (255)

60 (referral? adj3 (early or increase? or primary care or specialist? or general practitioner? or optimi?e? or optimal or reduce? or reducing)).ab. (4274)

61 referral?.ti. (7561)

62 (specialist? and (primary care or primary healthcare or gp or general practitioner? or family doctor)).ti. (504)

63 (specialist? adj3 (primary care or primary healthcare or gp or general practitioner? or family doctor)).ab. (1833)

64 reminder systems/ (1623)

65 guidelines as topic/ (25831)

66 guideline adherence/ or (guideline? adj3 (adherence or compliance or concordance or implement$ or uptake)).ti,ab. (18820)

67 "referral and consultation"/ (44723)

68 disease management/ (7435)

69 ("disease management" or "case management").ti. (4499)

70 patient-centered care/ or progressive patient care/ or patient care team/ (53023)

71 primary health care/ (44057)

72 critical pathways/ (3662)

73 delivery of health care, integrated/ (6411)

74 health services accessibility/ (39756)

75 managed care programs/ (22641)

76 product line management/ (1479)

77 health promotion/ (41316)

78 patient compliance/ (40116)

79 after-hours care/ (665)

80 (coordination or coordinated or multifactorial or multi-factorial or multicomponent or multi-component or multidisciplinary or multi-disciplinary or interdisciplinary or inter-disciplinary or integrated

or community-based or organized).ti. and ((care or approach or intervention or strategy or strategies or management or managing or center* or clinic*).ti. or "organization and administration"/) (13043)

81 total quality management/ or quality control/ (46116)

82 (quality and (continuous or total) and (management or improvement)).ti. (941)

83 (tqm or cqi).ti. (496)

84 (education and continuing and (medical or professional* or nursing or physician* or nurse*)).ti. (3662)

85 (outreach and (visit* or educational)).ti. (86)

86 (academic and detailing).ti. (74)

87 exp education, continuing/ (48492)

88 exp diffusion of innovation/ (12504)

89 (diffusion and (innovation or technology)).ti. (176)

90 exp medical audit/ (13090)

91 ((audit or feedback or compliance or adherence or training) and (improv$ or guideline$ or practice$ or medical or provider$ or physician$ or nurse$ or clinician$ or academic or visit$)).ti. or exp practice guidelines/ (77293)

92 ((financial or economic or physician$ or patient$) and incentive$).ti. or exp reimbursement mechanisms/ (27796)

93 exp medical informatics/ (249949)

94 (computer or (decision and support)).ti. (38462)

95 exp telemedicine/ or telemedicine.ti. or telecommunication$.ti. or exp internet/ or web.ti. or modem.ti. or telephone$.ti. or exp telephone/ (60617)

96 decision support systems, clinical/ or hospital information systems/ or medical order entry systems/ or drug therapy, computer-assisted/ or clinical pharmacy information systems/ or medical records systems, computerized/ (28971)

97 (decision and support).ti. (2230)

98 ((computeri?ed or computer or computer-based) and (order or entry)).ti. (497)

99 total quality management/ [ml] (11275)

100 (tqm or cqi).ti,ab. (1225)

101 (quality adj2 improv$).ti. (7352)

102 (quality adj improv$).ab. (9528)

103 total quality.ti,ab. (1258)

104 continuous quality.ti,ab. (1959)

105 continuous improvement.ti,ab. (997)

106 quality management.ti,ab. (3581)

107 management quality circles/ [ml] (1183)

108 (quality adj2 (collaborat$ or circle? or team? or group?)).ti,ab. (1874)

109 (("qi" or quality) adj2 (framework? or mapping or measure? or measuring or measurement?)).ti,ab. (9824)

110 ((system or systems) adj2 (improve? or improving or improvement?)).ti. (964)

111 ((system or systems) adj2 (improve? or improving or improvement?)).ab. (5434)

112 ((system or systems) adj2 (reengineer$ or re-engineer$ or redesign$ or re-design? or reinvent$ or re-invent$)).ti,ab. (294)

113 (improv$ adj2 (process or processes)).ti. (576)

114 (improv$ adj2 (process or processes)).ab. (3930)

115 ((process or processes) adj2 (reengineer$ or re-engineer$ or redesign$ or re-design$ or reinvent$ or re-invent$)).ti,ab. (427)

116 ("model for improv$" or "model to improve").ti,ab. (1630)

117 ((qi or qa or "quality assurance") adj2 (approach or collaborat$ or cycle? or measur$ or method$ or microsystem? or network? or practice? or procedure? or process$ or program$ or project? or rapid-cycle? or research or team? or tool?)).tw. (5497)

118 (pdsa or pdca or tqis or "plan do study" or "plan do check").ti,ab. (342)

119 ((shewhart or shewart or deming) adj3 (cycle or method$)).ti,ab. (68)

120 rapid cycle.ti,ab. (227)

121 (breakthrough adj3 (series or project or collaborative?)).ti,ab. (106)

122 (lean adj (approach or enterpri?e or management or method? or methodology or philosophy or practice or principles or program$ or thinking)).ti,ab. (106)

123 six sigma.ti,ab. (238)

124 ("concept map$" or "process map$" or "value stream map$" or "fault tree analysis" or "failure mode and effects analysis").ti,ab. (524)

125 ((business or e-business or corporat$) adj2 (approach$ or method$ or theor$)).ti,ab. (217)

126 (knowledge adj (application or broke$ or creation or diffus$ or disseminat$ or exchang$ or implement$ or management or mobili$ or translat$ or transfer$ or uptake or utili$)).ti,ab. or "knowledge translation"/ (1801)

127 (research$ adj (diffusion$ or disseminat$ or exchang$)).ti,ab. (111)

128 translational research.ti,ab. (2398)

129 ("research findings into action" or "research to action" or "research into action" or "evidence to action" or "evidence to practice").ti,ab. (5138)

130 (diffusion adj2 innovation).ti,ab. or diffusion of innovation/ [ml] (11311)

131 research utili?ation.ti,ab. (506)

132 ((knowledge or evidence) adj2 synthesis).ti,ab. (1291)

133 ((evidence or "evidence-base?") adj2 (translated or translating or translation or exchang$)).ti,ab. (555)

134 ((evidence or knowledge) adj2 synthesi$).ti,ab. (1752)

135 (evidence-base? adj2 (guideline? or protocol? or decision? or pathway? or policy or policies or treatment? or prevention or process$ or recommnedation? or algorithm? or practice or pharma$ or prescrib$)).ti,ab. (12468)

136 academic detailing.ti,ab. (255)

137 ("audit and feedback" or "self-audit$").ti,ab. (433)

138 (audit adj2 (quality or improvement? or healthcare or "health care" or "patient care" or policy or policies)).ti,ab. (510)

139 (barrier? and facilitator?).ti,ab. (1504)

140 (barrier? adj2 (change? or implementation)).ti,ab. (1245)

141 (booklet$ or brochure? or pamphlet? or paper-based or "printed material?").ti,ab. (6117)

142 (evidence-base? adj2 (policy$ or policies)).ti,ab. (495)

143 (program$ adj2 (effect$ or evaluat$ or introduc$ or impact)).ti,ab. (15543)

144 ((clinical or knowledge or evidence or quality or research or practice) adj2 gap?).ti,ab. (4270)

145 education, dental, continuing/ or education, medical, continuing/ or education, nursing, continuing/ or education, pharmacy, continuing/ or education, professional, retraining/ [ml] (41941)

146 (continuing adj2 education).ti,ab. (13314)

147 (retrain$ adj2 (doctor? or "health care" or nurse? or nursing or physician? or practitioner?)).ti,ab. (59)

148 workshop?.ti,ab. (21052)

149 inservice training/ or staff development/ (20719)

150 (professional development adj3 (doctor? or (("health care" or healthcare or hospital) adj (staff or worker? or employee?)) or nurse? or nursing or pharmacist$ or physician? or practitioner?)).ti,ab. (388)

151 guideline adherence/ [ml] (14331)

152 ((protocol? or guideline?) adj3 (adhere$ or concordance or effect$ or enforc$ or influenc$ or implement$ or impact$ or introduc$ or uptake)).ti,ab. (14497)

153 (incentiv$ adj2 (economic or employee? or financ$ or insurer? or insurance or market$ or monetar$ or pay$ or physician? or plan? or practitioner? or program$ or provider? or reimburs$ or salary or salarie? or staff or team$ or value-based)).ti,ab. (4252)

154 ("nurse-led" or "nurse-driven" or (nurse? adj2 (led or driven or managed or coordinat$ or co-ordinat$))).ti,ab. (2539)

155 (physician initiated or provider initiated or practitioner initiated).ti,ab. (236)

156 ((knowledge or evidence or practice) adj2 (gap? or barrier?)).ti,ab. (3863)

157 "opinion leader?".ti,ab. (606)

158 (outreach adj2 (communit$ or plan? or program? or visit?)).ti,ab. (1823)

159 ("physician$ behavio?r?" or "behavio?r? of physician?" or "nurse? behavio?r?" or "nursing behavio?r?" or "behavio?r? of nurse?" or "pharmacist? behavio?r?" or "behavio?r? of pharmacist?").ti,ab. (1761)

160 (practice pattern? adj3 (change$ or changing or effect? or impact? or improve$ or improving or modify$ or modifie? or moderate? or moderating)).ti,ab. (417)

161 ((policy$ or policies) adj2 (chang$ or effect? or impact? or influenc$)).ti,ab. (4931)

162 (quality adj2 (assurance or improvement? or initiativ$ or plan$ or program$ or review)).ti,ab. (34604)

163 (qi adj (inititative? or intervention? or program$ or plan$)).ti,ab. (189)

164 ((improvement or quality) adj2 (initiative? or program)).ab. (5193)

165 ((improv$ or effect?) adj2 performance).ti,ab. (23067)

166 (("user computer" or "computer user") adj4 (patient? or intervention? or care or healthcare)).ti,ab. [changed] (1)

167 computers, handheld/ or handheld?.ti,ab. or (pda or "personal data assistant?" or blackberr$).ti,ab. (7313)

168 (teleassist$ or tele-assist$ or teleaudiolog$ or tele-audiolog$ or telebased or tele-based or telecancer or tele-cancer or tele-cardiolo$ or telecardiolog$ or teleconsult$ or tele-consult$ or telecounselling or tele-counselling teledental or tele-dental or telederm$ or tele-derm$ or telediagnos$ or tele-diagnos$ or teledialysis or tele-dialysis or teleecho$ or tele-echo$ or teleemerg$ or tele-emerg$ or teleepileps$ or tele-epileps$ or telefollow$ or tele-follow$ or teleguidance or tele-guidance or telehealth$ or tele-health$ or telehome$ or tele-home$ or teleicu or tele-icu or teleintervention$ or tele-intervention$ or telemanag$ or tele-manag$ or telemedicine or tele-medicine or telemental$ or tele-mental$ or

telemonitor$ or tele-monitor$ or telenurs$ or tele-nurs$ or teleoncolo$ or tele-oncolo$ or teleopthalm$ or tele-opthalm$ or telepalliat$ or tele-palliat$ or tele-patholog$ or tele-patholog$ or teleprocedu$ or tele-procedu$ or telepsych$ or tele-psych$ or teleradiol$ or tele-radiol$ or telerefer$ or tele-refer$ or telerehab$ or tele-rehab$ or telesurger$ or tele-surger$ or telesurgic$ or tele-surgic$ or teletherap$ or tele-therap$ or teletreat$ or tele-treat$ or teletriage or tele-triage).ti,ab. (8950)

169 exp telemedicine/ or (telemedicine or telephone$ or cell-phone? or phone or phones).ti. (17789)

170 (e-health$ or e-medicine or e-practice).ti,ab. (760)

171 (video$ adj5 (diagnos$ or healthcare or "health care" or learning or "patient care" or teaching)).ti,ab. (1976)

172 (audiovisual aids/ or multimedia/) and (patient or inpatient?).ti,hw. (967)

173 social marketing/ or (marketing or "virtual communit$" or facebook or twitter or "social networking").ti,ab. (15488)

174 (effective adj2 (practice or healthcare or "health care")).ti,ab. (2042)

175 ((standard or usual) adj care adj3 (compar$ or change? or changing or improv$ or effect$ or enhanc$ or influenc$)).ti,ab. (1754)

176 ((doctor? or "healthcare provider?" or "health care provider?" or nurse? or nursing or physician? or practitioner?) adj2 (bonus$ or incentive? or reward?)).ti,ab. (617)

177 ((performance$ adj2 pay$) or p4p or "pay for quality improvement?" or p4qi or "fee-for service?").ti,ab. (4051)

178 (("performance based" or value-based) adj4 (salary or salaries or reimbursement? or pay$ or incentiv$ or physician? or healthcare or "health care" or "patient care" or hospital?)).ti,ab. (203)

179 ("hospital? performance" or "nurs$ performance" or "physician? performance").ti,ab. (1373)

180 (payment? adj2 (blend$ or "blue cross" or bonus$ or capitat$ or capped or "episode of care" or fixed or government$ or insur$ or level? or linear or medicaid or medicare or non-linear or per-patient or per-episode or per-visit or performance or prospectiv$ or retroactiv$ retrospectiv$ or reward$ or

schedule? or system? or target$ or third-part$ or threshold? or uncap$ or shared or variable or per-visit?)).ti,ab. (5563)

181 (non-compliance or noncompliance or nonadherence or non-adherence or nonpersistence or non-persistance or "inadequate adherence").ti,ab. (9762)

182 (change? or changing or improv$ or effect$ or influenc$).ti. and (health or care).hw. and "organization & administration".fs. (17625)

183 (change? or changing or improv$ or effect$ or influenc$).ti. and (hospital? or practice).hw. and "organization & administration".fs. (10304)

184 ((change? or changing or improv$ or effect$ or influenc$) adj2 ("healthcare delivery" or "health care delivery" or "delivery of health$" or (organi? adj2 healthcare) or (organi? adj2 "health care") or "patient outcome?" or "practic$ management" or provider? or "treatment outcome?" or "disease management")).ti,ab. (9526)

185 ((process or processes or system or systems) adj2 (improv$ or reengineer$ or re-engineer$ or redesign$)).ti,ab. (11175)

186 ((sub-optimal or suboptimal) adj2 (care or management or prescrib$)).ti,ab. (666)

187 (integrat$ adj2 (care or delivery or healthcare or "health care" or service?)).ti,ab. (6480)

188 ("patient? satisfaction" adj3 (improv$ or increas$)).ti,ab. (1933)

189 ((clinic$ or doctor? or patient? or physician?) adj2 referral?).ti,ab. (5099)

190 (((decreas$ or reduc$ or minimi$ or shorten or optimi$) adj3 "length of stay") or (wait adj2 time?)).ti,ab. (3078)

191 ((decreas$ or improv$ or reduc$ or minimi$ or shorten or optimi$) adj3 (readmission? or ((patient? or hospital?) adj4 (admission? or admitting or discharg$ or transfer?)))).ti,ab. (2789)

192 ((policy$ or policies) adj2 (chang$ or improv$ or implement$ or optimi$)).ti,ab. (6082)

193 (chang$ or effect$ or improv$ or implement$).ti. and (("health care" adj2 delivery) or (patient adj2 care) or (disease adj management) or (health$ adj2 service?) or (inpatient adj2 care) or (primary adj care) or policy or policies or "quality of health care").hw. (41002)

194 (chang$ or improv$ or effect$ or influenc$).ti. and ("health care" or care).hw. and "organization & administration".fs. (12019)

195 (performance adj improv$).ti,ab. (2991)

196 (blog$ or wiki$ or "web 2.0").ti,ab. (719)

197 (e-detailing or "electronic detailing").ti,ab. (6)

198 (web-based or internet-based or "digital technolog$").ti,ab. (11474)

199 (videoconferenc$ or video-conferenc$ or "video conferenc$" or web-cam$).ti,ab. (1268)

200 (voip or "voice over internet" or skype or "audiovisual dialog$" or "audio-visual dialog$").ti,ab. (43)

201 (wifi or "web conferenc$" or "instant messaging" or "instant messenger" or "online chat$" or (internet adj2 chat$) or roip or "radio over internet" or wideband).ti,ab. (1136)

202 (broadband adj2 (technology or technologies or network?)).ti,ab. (62)

203 (remote adj2 (access or clinic$ or diagnos$ or doctor? or learning or medicine or monitor$ or nurse? or nursing or patient or physician? or teaching)).ti,ab. (1618)

204 community-base?.ti. (6933)

205 collaborat$.ti. (15273)

206 team?.ti. (13987)

207 (virtual adj2 (class$ or communit$ or consult$ or diagnos$ or learning or medicine or reality or teaching)).ti,ab. (3529)

208 interdisciplinary communication/ or interdisciplinary.ti,ab. [ml] (21076)

209 ("cross-profession$" or intraprofession$ or intra-profession$ or (skill adj2 mix$) or teambase? or team-based).ti,ab. (1239)

210 interprofessional relations/ or interprofessional$.ti. (39139)

211 (multiintervention? or multi-intervention? or postintervention? or post-intervention? or preintervention? or pre-intervention?).ti,ab. (6262)

212 (teleassist$ or tele-assist$ or teleaudiolog$ or tele-audiolog$ or telebased or tele-based or telecancer or tele-cancer or tele-cardiolo$ or telecardiolog$ or teleconsult$ or tele-consult$ or telecounselling or tele-counselling teledental or tele-dental or telederm$ or tele-derm$ or telediagnos$ or tele-diagnos$ or teledialysis or tele-dialysis or teleecho$ or tele-echo$ or teleemerg$ or tele-emerg$ or teleepileps$ or tele-epileps$ or telefollow$ or tele-follow$ or teleguidance or tele-guidance or telehealth$ or tele-health$ or telehome$ or tele-home$ or teleicu or tele-icu or teleintervention$ or tele-intervention$ or telemanag$ or tele-manag$ or telemedicine or tele-medicine or telemental$ or tele-mental$ or telemonitor$ or tele-monitor$ or telenurs$ or tele-nurs$ or teleoncolo$ or tele-oncolo$ or teleopthalm$ or tele-opthalm$ or telepalliat$ or tele-palliat$ or tele-patholog$ or tele-patholog$ or teleprocedu$ or tele-procedu$ or telepsych$ or tele-psych$ or teleradiol$ or tele-radiol$ or telerefer$ or tele-refer$ or telerehab$ or tele-rehab$ or telesurger$ or tele-surger$ or telesurgic$ or tele-surgic$ or teletherap$ or tele-therap$ or teletreat$ or tele-treat$ or teletriage or tele-triage).ti,ab. (8950)

213 telemedicine/ or telenursing/ or remote consultation/ (10715)

214 (non-compliance or noncompliance or nonadherence or non-adherence or nonpersistence or non-persistance or "inadequate adherence").ti,ab. (9762)

215 or/2-214 [QI/Intervention terms] (1915913)

216 (systematic adj2 review).ti. (15975)

217 meta-analysis.pt. (28045)

218 (meta-analy* or metaanaly*).ti,ab. (35913)

219 (meta regression* or metaregression* or mega regression*).ti,ab. (1233)

220 ((systematic* or methodologic$) adj3 (review* or overview*)).ti,ab. (33103)

221 (quantitative adj3 (review* or overview* or synthes*)).ti,ab. (1743)

222 (research adj3 (integrati* or overview*)).ti,ab. (2234)

223 ((integrative or integrated) adj3 (review* or overview*)).ti,ab. (1067)

224 (collaborative adj3 review).ti,ab. (192)

225 "our review".ab. (3304)

226 (data synthes* or data extraction* or data abstraction*).ti,ab. (9434)

227 (pool$ adj3 analys$).ti,ab. (5260)

228 (mantel haenszel or peto or der simonian or dersimonian or fixed effect* or latin square*).ti,ab. (9617)

229 (medline or cochrane or pubmed or psychinfo or cinahl or medlars).ti,ab,hw,jn,jw. (55456)

230 (evidence adj3 (review? or appraisal? or overview* or synthesis)).ti,ab. (19226)

231 (critical adj3 (review? or appraisal? or overview?)).ti,ab. (14105)

232 ((synthes$ or evaluat$) adj2 evidence$).ti,ab. (4555)

233 randomized controlled trials as topic/ (72407)

234 meta-analysis as topic/ (11117)

235 or/216-234 [systematic review filter] (202958)

236 1 and 215 and 235 (1754)

237 (biography or case reports or clinical trial or clinical trial phase i or clinical trial phase ii or clinical trial phase iii or clinical trial phase iv or comparative study or controlled clinical trial or evaluation studies or guideline or in vitro or randomized controlled trial or validation studies).pt. (4043582)

238 (236 not (biography or case reports or clinical trial or clinical trial phase i or clinical trial phase ii or clinical trial phase iii or clinical trial phase iv or comparative study or controlled clinical trial or evaluation studies or guideline or in vitro or randomized controlled trial or validation studies)).pt. (1479)

239 limit 238 to yr="2009 -current" (471)

240 limit 238 to yr="1976-2008" (1008)

Database: Embase Classic+Embase <1947 to 2011 May 27>

5 *glycemic control/ (1694)

6 exp *diabetes mellitus/ or exp *diabetic angiopathy/ or exp *pregnancy diabetes mellitus/ (297546)

7 Diabet$.ti. (257458)

8 "systematic review"/ or meta-analysis/ or systematic review.ti. or (metaanaly$ or meta-analy$).ti. (86726)

9 (or/5-7) and 8 (2461)

10 limit 9 to yr="2009 -Current" (837)

11 (gene or genes or genetic? or genom$ or placebo).ti,hw. (2379819)

12 animal.ti,hw. (4265686)

13 10 not (or/11-12) (599)

Cochrane Library (Wiley)

#1 MeSH descriptor Diabetes Mellitus explode all trees 12988

#2 (diabet*):ti or (diabet*):kw in Cochrane Reviews 152

#3 #1 or #2 154
