# Supplementary material for: Improving quality of care for persons with diabetes: an overview of systematic reviews - what does the evidence tell us?
Source: Syst Rev. 2013 May 7;2:26. doi: 10.1186/2046-4053-2-26 (PMC3667096; doi:10.1186/2046-4053-2-26)
Supplement: Additional file 2 — Intervention categories identified. Description of the intervention categories used to classify reviews. [file 2046-4053-2-26-S2.pdf]

Additional file 2 – Intervention categories identified

Types of interventions

There are a variety of quality improvement and health system interventions that can be targeted at the patient, the healthcare provider, the healthcare system or multiple targets to improve the quality of diabetes care. We used the QI intervention categories of the McMaster Health Forum taxonomy[21] to organise the included reviews. The following are descriptions of four quality improvement categories that reviews were categorised to based on our assessment of overall intervention focus - including a description of a category we used to classify those reviews with a broad scope.

Broad based reviews – Reviews in this category evaluated a variety of different quality improvement and health system interventions to improve specific aspects of diabetes care (e.g., glycemic control) or to improve diabetes management and outcomes in specific patient populations (e.g., socially disadvantaged groups).

Organisational changes – Reviews in this category evaluated any intervention that involved a change in the structure or delivery of healthcare related to diabetes.

Patient education and support – Reviews in this category evaluated interventions that aimed to improve self-management by helping patients understand their treatment and their health in relation to diabetes. Interventions included those to educate, provide information, or promote health or treatment; those to train patients to correctly self-monitor, self-test, and administer treatment; those to provide emotional/personal support (such as individual, group, or family therapy); and those to support the adoption of health and treatment behaviours (such as reminders, goal-setting, and incentives).

Provider role changes – Reviews in this category evaluated the expansion of professional roles to include new tasks, substitution of a non-healthcare professional for a healthcare professional in the performance of a given task, and creation of multidisciplinary teams of healthcare workers. Expansion could, for example, involve pharmacists providing drug counselling that was formerly provided by nurses and physicians; nutritionists providing nursing care; or physical therapists providing nursing care. Substitution might include shifting tasks from physicians or nurses to community health workers.

Telemedicine – Reviews in this category assessed the effect of telephone, internet, and video communication between a patient and provider(s) or between providers. This could include communication and case discussion between distant health professionals, telephone support for patients, and patient-mediated telemonitoring (transmission of patient data to providers).
