# Supplementary material for: Improving quality of care for persons with diabetes: an overview of systematic reviews - what does the evidence tell us?
Source: Syst Rev. 2013 May 7;2:26. doi: 10.1186/2046-4053-2-26 (PMC3667096; doi:10.1186/2046-4053-2-26)
Supplement: Additional file 3 — Details of included reviews. Characteristics and results of the included reviews. [file 2046-4053-2-26-S3.pdf]

Additional file 3 – Details of included reviews

Lead Author, Year, Reference	Review Characteristics	Main Results†	Reported effect sizes and potential effect modifiers‡
**PATIENT EDUCATION AND SUPPORT**			
**Allemann 2009[26]**	**Study Design:** RCT<br>**Participants:** Patients, Providers<br>**Interventions:** Information or education provision, skills and competencies development, ICT that support individuals who receive care<br>**Search Dates:** Up to January 2009<br>**Focus:** Self-monitoring blood glucose (SMBG)	• 15 included studies (10 excluded from analysis)<br>**Glycemic Control (Clinical):**<br>• 5/5 RCTs favoured intervention for information or education provision<br>• 1/1 RCT favoured intervention for skills and competencies development<br>• 1/1 RCT favoured intervention for ICT that support individuals who receive care	• SMBG was associated with a significantly lower HbA1c compared with non-SMBG (WMD -0.31% (-0.44 to -0.17)).<br>• More frequent SMBG did not result in a significantly lower HbA1c compared with less intensive SMBG (WMD -0.21% (-0.57 to 0.15)).
**Armour 2005[27]**	**Study Design:** RCT<br>**Participants:** Patients, Other<br>**Interventions: Family targeted** (information or education provision, behaviour change support, personal support), **System targeted** (site of service delivery, skill mix- multidisciplinary teams, information or education provision)<br>**Search Dates:** Up to March 2003<br>**Focus:** Family interventions	• 19 included studies (5 excluded from analysis)<br>**Glycemic Control (Clinical):**<br>• 10/14 RCTs favoured intervention for multifaceted family targeted interventions<br>• 2/3 RCTs favoured intervention for multifaceted system targeted interventions	• There was a beneficial effect of family interventions on HbA1c for eight studies (-0.6 (-1.2 to -0.1)).
**Brown 1990[30]**<br>(companion paper)[29]	**Study Design:** RCT, CT, CBA, BA<br>**Participants:** Patients<br>**Interventions:** Information or education provision, behaviour change support, skills and competencies development<br>**Search Dates:** From 1961 up to 1989<br>**Focus:** Patient education	• 82 included studies (45 excluded from analysis)<br>**Glycemic Control (Clinical):**<br>• 27/30 studies favoured intervention for information or education provision (10/12 RCTs)<br>• 1/4 studies favoured intervention for behaviour change support (0/1 RCT)	• The authors reported that weighted effect size estimates were in the moderate range for glycosylated hemoglobin (0.41 (0.31 to 0.52), n=27), cholesterol (0.24 (0.09 to 0.38), n=9) and blood pressure (0.34 (0.14 to 0.55), n=3).

Lead Author, Year, Reference	Review Characteristics	Main Results†	Reported effect sizes and potential effect modifiers‡
		• 1/1 RCT favoured intervention for skills and competencies development<br>**Vascular Risk Factors (Clinical):**<br>**Cholesterol:**<br>• 8/8 studies favoured intervention for information or education provision (3/3 RCTs)<br>• 1/1 study favoured intervention for behaviour change support<br>**Blood Pressure:**<br>• 2/2 studies favoured intervention for information or education provision<br>• 1/1 study favoured intervention for skills and competencies development	
**Cooper 2009[34]**	**Study Design:** RCT<br>**Participants:** Patients<br>**Interventions:** ICT that support individuals who receive care, behaviour change support, information or education provisions, personal support, patient mediated, educational meetings<br>**Search Dates:** Unspecified<br>**Focus:** Technology based approaches to patient education for young people with diabetes	• 5 studies representative of 11 papers (4 excluded from analysis)<br>**Glycemic Control (Clinical):**<br>• 1/1 RCT favoured intervention	No meta-analysis or meta-regression.
**Couch**	**Study Design:** RCT, CCT, BA,	• 80 included studies (45 excluded	No meta-analysis or meta-regression.

Lead Author, Year, Reference	Review Characteristics	Main Results†	Reported effect sizes and potential effect modifiers‡
**2008[35]**	CBA, other<br>**Participants:** Patients, Providers, Other<br>**Interventions:** Information or education provisions, skills and competencies development, personal support, behaviour change support, self-management<br>**Search Dates:** From 1982 up to March 2007<br>**Focus:** Patient and family education	from analysis)<br>**Glycemic Control (Clinical):**<br>• 27/35 studies favoured intervention (16/23 RCTs)	
**Deakin 2005[36]**	**Study Design:** RCT, CT<br>**Participants:** Patients, Providers, Other<br>**Interventions: Information or education provision**, self-management, skill mix - multidisciplinary teams, skill mix - role expansion or substitution<br>**Search Dates:** Up to January and February 2003<br>**Focus:** Group-based education	• 11 included studies (0 excluded from analysis)<br>**Glycemic Control (Clinical):**<br>• 11/11 studies favoured intervention (8/8 RCTs)<br>**Vascular Risk Factors (Clinical):**<br>**Cholesterol:**<br>• 0/3 RCTs favoured intervention for total cholesterol<br>**Blood Pressure:**<br>• 5/5 studies favoured intervention (3/3 RCTs)	• Interventions improved glycemic control (HbA1c levels) at all time points (4 to 6mths: MD -1.35 (-1.93 to -0.78); 12 to 14mths: MD -0.82 (-0.99 to -0.65); 2yrs: MD -0.97 (-1.40 to -0.54)).<br>Improvements in vascular risk factors were also associated with this intervention (SBP 4-6mths: MD -5.37 (-9.53 to -1.21); 12-14mths: MD -2.61 (-6.74 to 1.5); DBP 4-6mths: MD -2.65 (-5.57 to 0.28)).
**Duke 2009[37]**	**Study Design:** RCT<br>**Participants:** Patients, Providers, Other (not-specified)<br>**Interventions:** Information or education provision, personal support, behaviour change support<br>**Search Dates:** Up to April 2007<br>**Focus:** Individual education	• 9 included studies (0 excluded from analysis)<br>**Glycemic Control (Clinical):**<br>• 5/9 RCTs favoured intervention<br>**Vascular Risk Factors (Clinical):**<br>**Cholesterol:**<br>• 2/4 RCTs favoured intervention<br>**Blood Pressure (Diastolic):**<br>• 2/5 RCTs favoured intervention<br>**Blood Pressure (Systolic):**<br>• 2/5 RCTs favoured intervention	• For individual education versus usual care there was no significant difference at 12 to 18 months in either HbA1c (WMD -0.1% (-0.3 to 0.1)), total cholesterol (WMD -0.03 mmol/L (-0.2 to 0.10)), systolic blood pressure (WMD -2 mm Hg (-5 to 1)), or diastolic blood pressure (WMD -2mmHg (-3 to 0.00)).<br>• For individual education versus group education there was no significant

Lead Author, Year, Reference	Review Characteristics	Main Results†	Reported effect sizes and potential effect modifiers‡
		**Smoking Cessation (Clinical):**<br>• 0/2 RCTs favoured intervention	difference between the interventions at 12 to 18 months in HbA1c (WMD 0.03% (-0.02 to 0.1)), systolic blood pressure (WMD 4 mm Hg (-4 to 12)), or diastolic blood pressure (WMD 2 mm Hg (-4 to 7)).<br>• There was no significant difference between individual education and group education in change in total cholesterol over 3, 6 or 12 months (n=1).
**Ellis 2004[38]**	**Study Design:** RCT<br>**Participants:** Patients, Providers<br>**Interventions: Behaviour change support**, information or education provision, staff/self shared decision making, personal support<br>**Search Dates:** Up to December 2000<br>**Focus:** Patient education	• 21 included studies (0 excluded from analysis)<br>**Glycemic Control (Clinical):**<br>• 27/28 RCTs favoured intervention	Meta-regression revealed that several attributes of patient education may predict improved glycemic control: face-to-face interaction -1.45 (-1.87 to -1.02); a cognitive reframing teaching method 2.34 (3.93 to 0.74); and exercise content -1.51 (-2.01 to - 1.01).
**Gary 2003[42]**	**Study Design:** RCT<br>**Participants:** Providers, Patients<br>**Interventions:** Information or education provision, reminders and prompts<br>**Search Dates:** Up to 1999<br>**Focus:** Self-care behaviours	• 18 included studies (2 excluded from analysis)<br>**Glycemic Control (Clinical):**<br>• 13/15 RCTs favoured intervention for information or education provision<br>• 1/1 RCT favoured intervention for reminders and prompts	• Most interventions produced a decline in HbA1c compared with controls. The pooled effect size (SMD) was -0.43% (p=0.003).<br>• Stratified analyses were conducted for pooled effect sizes for HbA1c based on the most frequent intervention characteristics: interventionist, mode of instruction and topic of instruction.<br>• Effect sizes were -1.80% for studies that used physicians (p=0.229), -0.71% for studies that used nurses (p=0.022), and -0.88% for studies that used dieticians (p=0.043).<br>• Studies with individual or group

Lead Author, Year, Reference	Review Characteristics	Main Results†	Reported effect sizes and potential effect modifiers‡
			modes of instruction produced similar effect sizes: -0.62% (p=0.005) and -0.70% (p=0.015), respectively. Studies with topic areas focusing on medications had the largest effect size (-0.72%; p=0.032), followed by exercise (-0.69%; p=0.007); diet (-0.51%; p=0.008), and blood glucose self-monitoring (-0.20%; p<0.001).
**Hampson 2001[45]** (companion paper) [41]	**Study Design:** RCT, CT, BA, Other<br>**Participants:** Patients, Family, Providers, Other<br>**Interventions:** Behaviour change support, personal support, information or education provision, skills and competencies development, ICT that supports individuals who receive care, site of service delivery<br>**Search Dates:** Up to June 1999<br>**Focus:** Education on disease management	• 64 included studies (35 excluded from analysis)<br>**Glycemic Control (Clinical):**<br>• 7/10 studies favoured intervention for behaviour change support (2/5 RCTs)<br>• 5/5 studies favoured intervention for personal support (1/1 RCTs)<br>• 5/5 studies favoured intervention for information or education provision (1/1 RCT)<br>• 3/4 RCTs favoured intervention for skills and competencies development<br>• 1/2 studies favoured intervention for ICT that supports individuals who receive care (0/1 RCTs)<br>• 1/1 RCT favoured intervention for site of service delivery<br>**Vascular risk factors (Clinical):**<br>**Cholesterol:**<br>• 1/1 study favoured intervention for personal support<br>• 1/1 study favoured intervention for information or education provision	• The mean of the 12 effect sizes for HbA1c was 0.33 (-0.04 to 0.70).

Lead Author, Year, Reference	Review Characteristics	Main Results†	Reported effect sizes and potential effect modifiers‡
**Harkness 2010[46]**	**Study Design:** RCT<br>**Participants:** Patients, Providers<br>**Interventions:** Behaviour change support, information or education provisions, skills and competencies development, personal support, case management<br>**Search Dates:** Up to April 2009<br>**Focus:** Psychosocial interventions that improve both physical and mental health in patients with diabetes	• 49 included studies (0 excluded from analysis)<br>**Glycemic Control (Clinical):**<br>• 12/13 RCTs favoured behaviour change support intervention (one study reported no difference between intervention and control)<br>• 6/7 RCTs favoured behaviour change support, personal support and other (skills and competencies, case management) interventions<br>• 14/14 RCTs favoured behaviour change support, information or education provision, skills and competencies, and personal support interventions<br>• 9/11 RCTs favoured information or education provisions and skills and competencies interventions (two studies reported no difference between intervention and control)<br>• 1/3 RCTs favoured personal support intervention (one study reported no difference between intervention and control)<br>• 1/1 RCT favoured skills and competencies intervention	• Psychosocial interventions modestly improved HbA1c (SMD -0.29 (-0.37 to -0.21)).<br>• Authors note that interventions focused on lifestyle alone were not significantly more effective in controlling HbA1c than those focused on mental health or combined interventions.<br>• The benefits of psychosocial interventions on HbA1c were less in elderly patients (mean age >50 years) (0.16 (-0.02 to 0.34)), and greater in those recruited on the basis of poor baseline diabetes control (-0.17 (-0.37 to 0.03)).
**Hawthorne 2008[47]**	**Study Design:** RCT<br>**Participants:** Patients, Providers, Other<br>**Interventions: Culturally appropriate care, information or education provision**, behaviour change support	• 11 included studies (1 excluded from analysis)<br>**Glycemic Control (Clinical):**<br>• 7/10 RCTs favoured intervention<br>**Vascular risk factors (Clinical):**<br>**Cholesterol:**<br>• 5/7 RCTs favoured intervention	• Culturally appropriate education improved glycemic control (HbA1c levels) at three (WMD -0.3% (-0.6 to -0.01)) and six months (WMD -0.6% (-0.9 to -0.4)) but had no effect at 12 months (WMD -0.1% (-0.4 to 0.2)).

Lead Author, Year, Reference	Review Characteristics	Main Results†	Reported effect sizes and potential effect modifiers‡
	**Search Dates:** Up to August 2007<br>**Focus:** Culturally appropriate education	**Blood Pressure:**<br>• 2/4 RCTs favoured intervention	Interventions had no significant effect on systolic or diastolic blood pressure at any time point. Total cholesterol levels at one year showed an improvement in the intervention groups (WMD -0.39 g/dl (-0.64 to -0.14)).
**Loveman 2003[52]**	**Study Design:** RCT, CCT<br>**Participants:** Patients, Providers, Other<br>**Interventions: Information or education provision**, behaviour change support, skills and competencies development, culturally appropriate care<br>**Search Dates:** Up to 2002<br>**Focus:** Clinical- and cost-effectiveness of educational interventions	• 24 included studies (1 excluded from analysis)<br>**Glycemic Control (Clinical):**<br>• 20/23 studies favoured intervention (14/17 RCTs)<br>**Vascular risk factors (Clinical):**<br>**Cholesterol:**<br>• 7/9 studies favoured intervention (6/8 RCTs)<br>**Blood Pressure:**<br>• 5/5 studies favoured intervention (4/4 RCTs)	No meta-analysis or meta-regression.
**Loveman 2008 [53]**	**Study Design:** RCT, CCT<br>**Participants:** Patients, Providers<br>**Interventions: Information or education provision**, behaviour change support, skills and competencies development, culturally appropriate care<br>**Search Dates:** 2002 up to January 2007<br>**Focus:** Clinical- and cost-effectiveness of educational interventions	• 21 included studies (1 excluded from analysis)<br>**Glycemic Control (Clinical):**<br>• 19/24 studies favoured intervention (15/20 RCT comparisons)<br>**Vascular risk factors (Clinical):**<br>**Cholesterol:**<br>• 6/11 studies favoured intervention (4/9 RCT comparisons)<br>**Blood Pressure (Systolic)**<br>• 5/5 RCTs favoured intervention<br>**Blood Pressure (Diastolic)**<br>• 6/7 studies favoured intervention (4/5 RCT comparisons)	No meta-analysis or meta-regression.
**Minet**	**Study Design:** RCT	• 47 included studies (0 excluded	• Meta-analysis showed a 0.36% (0.21

Lead Author, Year, Reference	Review Characteristics	Main Results†	Reported effect sizes and potential effect modifiers‡
**2010[55]**	**Participants:** Patient, Provider<br>**Interventions: Educational intervention** (information or education provisions, skills and competencies development); **Behavioural psychosocial intervention** (behaviour change support, personal support)<br>**Search Dates:** Up to November 30, 2007<br>**Focus:** Self-care management interventions in type 2 diabetes	from analysis)<br>**Glycemic Control (Clinical):**<br>• 24/29 RCTs favoured educational intervention<br>• 12/16 RCTs favoured behavioural psychosocial intervention<br>• 1/2 RCTs favoured a combination of educational and behavioural psychosocial intervention	to 0.51) improvement in glycemic control in people who received self-care management treatment.<br>• Regression analyses showed a non-significant 0.26% (p=0.107) larger reduction in HbA1c in studies using educational techniques compared to studies using behavioural psychosocial techniques.
**Norris**<br>**2001 [58]**	**Study Design:** RCT<br>**Participants:** Patients, Providers, Other<br>**Interventions:** Behaviour change support, skills and competencies development, information or education provision<br>**Search Dates:** 1980 to December 1999<br>**Focus:** Self-management training	• 72 included studies (48 excluded from analysis)<br>**Glycemic Control (Clinical):**<br>• 5/7 RCTs favoured intervention for behaviour change support<br>• 5/7 RCTs favoured intervention for skills and competencies development<br>• 1/1 RCT favoured intervention for behaviour change support combined with skills and competencies development combined<br>• 1/1 RCT favoured intervention for information or education provision<br>**Vascular risk factors (Clinical):**<br>**Cholesterol:**<br>• 5/5 RCTs favoured intervention for behaviour change support<br>• 2/3 RCT favoured intervention for skills and competencies development	No meta-analysis or meta-regression.

Lead Author, Year, Reference	Review Characteristics	Main Results†	Reported effect sizes and potential effect modifiers‡
		**Blood pressure (Diastolic):**<br>• 0/1 RCTs favoured intervention for behaviour change support<br>• 2/2 RCT favoured intervention for behaviour change support combined with skills and competencies development<br>**Blood Pressure (Systolic):**<br>• 1/1 RCT favoured intervention for behaviour change support combined with skills and competencies development<br>**Foot Screening:**<br>• 1/1 RCT favoured intervention for skills and competencies development	
**Norris 2002[59]**	**Study Design:** RCT, CCT, BA, Other<br>**Participants:** Patients, Family, Providers, Other<br>**Interventions: Community** (**information or education provision**, skills and competencies development), **Home** (**information or education provision**, skills and competencies development, behaviour change support, ICT that support individuals who receive care), **Recreational Camps** (**information or education provision**, behaviour change support), **Worksite** (information or education provision)	• 30 included studies (13 excluded from analysis)<br>**Glycemic Control (Clinical):**<br>• 6/6 studies favoured intervention for community interventions (3/3 RCTs)<br>• 5/6 studies favoured intervention for home interventions (4/5 RCTs)<br>• 2/3 studies favoured intervention for recreational camp interventions (1/2 RCTs)<br>• 1/1 study favoured intervention for worksite interventions<br>**Vascular risk factors (Clinical):**<br>**Cholesterol:**<br>• 1/3 studies favoured intervention for community interventions	• Self-management education improved HbA1c levels in patients when delivered in the community (pooled estimate: -1.9 (-2.4 to -1.4)), in the home (pooled estimate type 1: -1.1 (-1.6 to -0.6); type 2: -0.5 (-1.1 to -0.1)). Absolute changes calculated for two studies indicated mixed results for the effectiveness of self-management education provided at recreational camps (-1.8% and 0.3% respectively).<br>• Community delivered interventions also improved total cholesterol (-2.6mg/dL (-54.0 to 6.0)), and blood pressure as reported for two studies (mmHg absolute change for systolic blood pressure: -12.3 and -8.6

Lead Author, Year, Reference	Review Characteristics	Main Results†	Reported effect sizes and potential effect modifiers‡
	**Search Dates:** Up to December 2000<br>**Focus:** Self-management education	**Blood Pressure:**<br>• 2/2 studies favoured intervention for community interventions	respectively, for diastolic blood pressure: -5.2 and -1.0 respectively).
**Norris 2005 [60]**	**Study Design:** RCT, CT<br>**Participants:** Patients, Providers<br>**Interventions:** Behaviour change support, personal support, skills and competencies development, information or education provision<br>**Search Dates:** Up to May 2004<br>**Focus:** Weight loss and weight control	• 24 included studies (15 excluded from analysis)<br>**Glycemic Control (Clinical):**<br>• 5/9 studies favoured intervention (5/8 RCTs)<br>**Vascular risk factors (Clinical):**<br>**Cholesterol:**<br>• 2/3 RCTs favoured intervention<br>**Blood Pressure (Systolic):**<br>• 2/2 RCTs favoured intervention<br>**Blood Pressure (Diastolic):**<br>• 0/2 RCTs favoured intervention	• Between-group pooled estimates of HbA1c were generally not significant, although several included studies did have a significant decrease (n=4). Between-study heterogeneity was significant ($p<0.05$) for these small groups of studies.<br>• Systolic blood pressure was examined in six studies, with a between-group change ranging between 1 mmHg and -4 mmHg; similar results were noted for diastolic blood pressure. Thirteen studies reported between-group change in total cholesterol (range, -0.4mmol/L (-7.2mg/dL) to 0.3mmol/L (5.9 mg/dL)).
**Savage 2010[65]**	**Study Design:** RCT<br>**Participants:** Patients, Providers, Other<br>**Interventions:** Information or education provision, behaviour change support, personal support, skills and competencies development, ICT that support individuals who receive care<br>**Search Dates:** January 1, 2004 to December 31, 2008<br>**Focus:** Interventions directed at children and/or adolescents (up to 18 years of age) with type 1 diabetes	• 14 included studies (in 29 papers) (8 excluded from analysis)<br>**Glycemic Control (Clinical)**<br>• 5/6 RCTs favoured intervention	No meta-analysis or meta-regression.

Lead Author, Year, Reference	Review Characteristics	Main Results†	Reported effect sizes and potential effect modifiers‡
**Valk 2001[69]**	**Study Design:** RCT<br>**Participants:** Patients, Providers<br>**Interventions:** Information or education provision, skills and competencies development<br>**Search Dates:** Up to September 2004<br>**Focus:** Foot care education	• 9 included studies (5 excluded from analysis)<br>**Foot Screening:**<br>• 6/9 RCTs favoured intervention for foot outcomes (ulcerations, infections, amputations)	No meta-analysis or meta-regression.[a]
**Winkley 2006[72]**	**Study Design:** RCT<br>**Participants:** Patients, Providers<br>**Interventions: Children (personal support**, behaviour change support, information or education provision), **Children** (behaviour change support), **Adults** (personal support), **Adults** (behaviour change support)<br>**Search Dates:** Up to September 2004<br>**Focus:** Psychological therapies	• 29 included studies (8 excluded from analysis)<br>**Glycemic Control (Clinical):**<br>• 3/6 RCTs favoured intervention for personal support for children<br>• 3/4 RCTs favoured intervention for behaviour change support for children<br>• 3/4 RCTs favoured intervention for personal support for adults<br>• 5/7 RCTs favoured intervention for behaviour change support for adults	• There were 10 studies in children and adolescents (n=543 participants) and 11 in adults (n=516 participants) with data that could be pooled.<br>• With random effects meta-analyses, there was a small to moderate pooled estimate of the mean standardised effect sizes (-0.35 (-0.66 to -0.04)) combined across all studies in children, but this association was attenuated when the authors combined data across all studies in adults (-0.17 (-0.45 to 0.10)). The standardised effects translated into absolute reductions in HbA1c of 0.48% (0.05% to 0.91%) for children and adolescents and of 0.22% (-0.13% to 0.56%) for adults.
**TELEMEDICINE INTERVENTIONS**			
**Balas 2004[28]**	**Study Design:** RCT<br>**Participants:** Patients, Providers<br>**Interventions: Reminders and prompts**, **Home glucose monitoring** (ICT that support individuals who receive care),	• 44 included studies (19 excluded from analysis)<br>**Glycemic Control (Process):**<br>• 3/3 RCTs favoured intervention for reminder and prompts<br>**Glycemic Control (Clinical):**	• The authors' meta-analysis of 16 studies in which home glucose records were used to perform computer assisted insulin dose adjustment resulted in significant decrease in HbA1c (average decrease of 0.14

Lead Author, Year, Reference	Review Characteristics	Main Results†	Reported effect sizes and potential effect modifiers‡
	**Transmission of home glucose records** (ICT that support individuals who receive care, patient-mediated), **Computerised education** (ICT that support individuals who receive care, information or education provision, behaviour change support)<br>**Search Dates:** Not specified<br>**Focus:** Automated or computerised information interventions	• 5/8 RCTs favoured intervention for home glucose monitoring<br>• 10/11 RCTs favoured intervention for transmission of home glucose records<br>• 2/3 RCTs favoured intervention for computerised education<br>**Retinopathy Screening:**<br>• 1/1 RCT favoured intervention for reminders and prompts<br>**Foot Screening:**<br>• 1/1 RCT favoured intervention for reminders and prompts	(0.11 to 0.16)).
**Farmer 2005[39]**	**Study Design:** RCT, CT, Other<br>**Participants:** Patients, Providers<br>**Interventions:** ICT that support individuals who receive care, patient mediated<br>**Search Dates:** Up to July 2004<br>**Focus:** Telemedicine for glycemic control	• 26 included studies (16 excluded from analysis)<br>**Glycemic Control (Clinical):**<br>• 6/10 studies favoured intervention (5/9 RCTs)	Pooled analysis revealed a non-significant reduction in HbA1c (-0.1% (-0.4 to 0.04)).
**Liang 2011[49]**	**Study Design:** RCT, CT, CBA<br>**Participants:** Patients, Providers<br>**Intervention: ICT that support individuals who receive care**, patient mediated, behaviour change support, information or education provisions, skills mix – multidisciplinary team, personal support<br>**Search Dates:** January 1990 to February 2010<br>**Focus:** Mobile phone interventions	• 22 included studies (0 excluded from analysis)<br>**Glycemic Control (Clinical):**<br>• 11/12 studies favoured intervention using SMS and internet (8/9 RCTs)<br>• 8/10 studies favoured intervention using SMS alone (4/6 RCTs)	• Random effects meta-analysis of the 22 trials involving mobile phone intervention showed an overall reduction in HbA1c by 0.51% (0.69% to 0.33%).<br>• Subgroup meta-analysis showed that interventions using SMS and internet resulted in a 0.7% decrease (0.4% to 0.9%), while SMS alone resulted in a 0.4% decrease (0.1% to 0.6%).
**Montori**	**Study Design:** RCT	• 8 included studies (0 excluded from	• Telecare interventions were not

Lead Author, Year, Reference	Review Characteristics	Main Results†	Reported effect sizes and potential effect modifiers‡
**2004[56]**	**Participants:** Patients, Providers<br>**Interventions: Transmission of home glucose readings** (ICT that support individuals who receive care, patient-mediated), information or education provision<br>**Search Dates:** From 1982 up to June 2003.<br>**Focus:** Telecare (transmission of blood glucose readings)	analysis)<br>**Glycemic Control (Clinical):**<br>• 5/7 RCTs favoured intervention for transmission of home glucose readings<br>• 1/1 RCT favoured intervention for information or education provision	significantly better than control interventions at improving glycemic control (pooled HbA1c change from baseline: 0.2% (-0.2% to 0.6%)), though a small positive effect favouring the intervention is indicated.
**Polisena 2009[62]**	**Study Design:** RCT, CT, BA<br>**Participants:** Patients, Providers, System<br>**Interventions:** ICT that support individuals who receive care, personal support<br>**Search Dates:** 1998 to 2008<br>**Focus:** Home telehealth and telephone support	• 26 included studies (8 excluded from analysis)<br>**Glycemic Control (Clinical):**<br>• 8/13 studies favoured intervention for ICT that support individuals who receive care (7/11 RCTs)<br>• 3/5 RCTs favoured intervention for personal support	• Home telemonitoring was significantly better than usual care at improving glycemic control, as measured by HbA1c (WMD -0.22 (-0.35 to -0.08)).
**Russell-Minda 2009[64]**	**Study Design:** RCT<br>**Participants:** Patients, Provider<br>**Interventions: ICT that support individuals who receive care**, **patient mediated**, personal support, information and education, skills and competencies, behaviour change support, educational materials<br>**Search Date:** 1985 to 2008<br>**Focus:** Health technologies for monitoring and managing diabetes	• 19 comparisons in 18 included studies (9 excluded from analysis)<br>**Glycemic Control (Clinical):**<br>• 10/10 RCTs favoured intervention (2 studies showed no difference between intervention and control)	• No meta-analysis or meta-regression.
**Shulman**	**Study Design:** RCT	• 10 included studies (0 excluded	• Meta-analysis concluded telemedicine

Lead Author, Year, Reference	Review Characteristics	Main Results†	Reported effect sizes and potential effect modifiers‡
**2010[67]**	**Participants:** Patients, Providers<br>**Interventions: ICT that support individuals who receive care, patient mediated, personal support,** information or education provisions<br>**Search Date:** Up to Dec 21, 2009<br>**Focus:** Impact of telemedicine interventions in youth with type 1 diabetes	from analysis)<br>**Glycemic Control (Clinical)**<br>• 4/6 RCTs favoured the interventions ICT that support individuals who receive care, patient mediated and personal support (2 studies had unclear results)<br>• 2/4 RCTs favoured the interventions ICT that support individuals who receive care, patient mediated, personal support and information or education provisions (1 study had unclear results)	decreased HbA1c levels (-0.12 (-0.35 to 0.11)) (based on nine studies).<br>• The pooled estimate for a between-study analysis comparing studies with a mean baseline HbA1c value <9% to those >9% trended towards favouring telemedicine in the subgroup of studies with the baseline HbA1c >9% (-0.25 (-0.55 to 0.04) for >9%; 0.07 (-0.29 to 0.44) for <9%).
**Sutcliffe 2011[68]**	**Study Design:** RCT, BA, COT, other<br>**Participants:** Patients, Provider, Other<br>**Interventions: Communication between patient and provider as facilitated by technology** (ICT that support individuals who receive care, patient mediated), skill mix – role expansion or extension (nurse or pharmacist-led), personal support, behaviour change support, information or education provisions<br>**Search Dates:** January 1990 to May 2009<br>**Focus:** Communication technology involving feedback between patient and provider	• 19 included studies (6 excluded from analysis)<br>**Glycemic Control (Clinical):**<br>• 6/8 studies favoured interventions using novel electronic communication (5/6 RCTs)<br>• 1/1 RCT favoured interventions using mobile telephony (SMS)<br>• 1/3 studies favoured interventions using telephone support (0/2 RCTs)<br>• 1/1 study favoured interventions using video- and tele-conferencing	• No meta-analysis or meta-regression.

Lead Author, Year, Reference	Review Characteristics	Main Results†	Reported effect sizes and potential effect modifiers‡
**Verhoeven 2007[70]**	**Study Design:** RCT<br>**Participants:** Patients, Carers, Providers, System<br>**Interventions: Patient transmission of clinical information** (ICT that support individuals who receive care, patient-mediated, personal support, information or education provision, reminders and prompts, behaviour change support), **Data interchange** (other ICT that support individuals who provide care, electronic health records), **Videoconference** (skill mix - communication and case discussion between distant health professionals), **Pharmacist-led education** (information or education provision)<br>**Search Dates:** 1994 to 2006<br>**Focus:** Teleconsultation and videoconferencing	• 39 included studies (20 excluded from analysis)<br>**Glycemic Control (Clinical):**<br>• 9/15 studies favoured intervention for patient transmission of clinical information interventions (4/10 RCTs)<br>• 2/2 studies favoured intervention for data interchange interventions<br>• 1/1 study favoured intervention for videoconferencing interventions<br>• 1/1 study favoured intervention for pharmacist-led education interventions<br>**Vascular Risk Factors (Clinical):**<br>**Cholesterol:**<br>• 1/1 study favoured intervention for data interchange interventions<br>**Blood Pressure:**<br>• 1/1 study favoured intervention for data interchange interventions<br>• 1/1 study favoured intervention for videoconferencing interventions	• The authors conducted a meta-analysis on the effects of teleconsultation on HbA1c values. The pooled reduction in HbA1c was not statistically significant (WMD -0.03 (-0.31 to 0.24)).
**Wu 2010[73]**	**Study Design:** RCT<br>**Participants:** Patients, Providers<br>**Interventions: ICT that support individuals who receive care**, behaviour change support, information or education provisions, skills and competencies development, personal support<br>**Search Dates:** 1950 to 2010<br>**Focus:** Telephone follow up to improve glycemic control in	• 7 included studies (0 excluded from analysis)<br>**Glycemic Control (Clinical):**<br>• 5/7 RCTs favoured intervention (1 study showed no difference between intervention and control)	• Pooled meta-analysis favoured telephone follow-up intervention with a WMD of -0.44 (-0.93 to 0.06), p=0.08.<br>• Subgroup analysis shows more intensive interventions (interactive follow up plus automated or non-interactive follow up) showed a significant benefit in favour of the treatment group, with a SMD of -0.84 (-1.67 to 0.0), p=0.05.

Lead Author, Year, Reference	Review Characteristics	Main Results†	Reported effect sizes and potential effect modifiers‡
	patients with Type 2 diabetes		
**PROVIDER ROLE CHANGES**			
**Alam 2009[25] (**companion paper) [48]	**Study Design:** RCT<br>**Participants:** Patients, Providers<br>**Interventions: Generalist** (behaviour change support, information or education provision), **Specialist** (behaviour change support, information or education provision)<br>**Search Dates:** Up to March 2007<br>**Focus:** Psychological interventions delivered by generalists versus specialists	• 35 included studies (16 excluded from analysis)<br>**Glycemic Control (Clinical):**<br>• 7/9 RCTs favoured intervention for generalist-led intervention<br>• 6/9 RCTs favoured intervention for specialist-led intervention<br>• 1/1 RCTs favoured intervention with unspecified leader	• For psychological interventions overall, the pooled effect size for HbA1c was -0.32 (-0.47 to -0.16).<br>• The delivery of the intervention by psychological specialists (n=9) improved HbA1c levels (SMD: -0.36 (-0.61 to -0.12)). Delivery of the intervention by generalist clinicians (n=9) also improved HbA1c levels (SMD: -0.27 (-0.50 to -0.04)).<br>• Specialist providers appear to be more effective than generalist providers in improving glycemic control.<br>• Sub-group analysis found evidence of an association between improvements in HbA1c and increased number of sessions (regression coefficient=0.04, p=0.001).
**Clark 2011[33]**	**Study Design:** RCT<br>**Participants:** Patients, Providers<br>**Interventions: Skill mix – role expansion or extension (nurse led care)**, **skill mix – multidisciplinary team**, educational materials (treatment algorithms), staff - altering the work load, culturally appropriate care, patient education (information or education provisions, behaviour change support, personal support)	• 11 included studies (3 excluded from analysis)<br>**Vascular Risk Factors (Clinical):**<br>**Systolic Blood Pressure:**<br>• 5/6 RCTs favoured interventions that are nurse-led<br>• 2/2 RCTs favoured interventions that are led by a multidisciplinary team<br>**Diastolic Blood Pressure:**<br>• 4/6 RCTs favoured interventions that are nurse-led<br>• 1/1 RCT favoured interventions are	• Meta-analysis of systolic blood pressure was -2.30 mmHg for five studies (-4.9 to 0.4).<br>• Meta-analysis of diastolic blood pressure was -0.95 mmHg for five studies (-2.75 to 0.84).<br>• Meta-analysis of rates of use of blood pressure medication was RR 1.02 for five studies (0.97 to 1.07).

Lead Author, Year, Reference	Review Characteristics	Main Results†	Reported effect sizes and potential effect modifiers‡
	**Search Dates:** 2002 up to November 2009<br>**Focus:** Nurse led interventions for the control of hypertension	led by a multidisciplinary team<br>**Use of Antihypertensive Medicine:**<br>• 5/5 studies were unclear in their results	
**Lindenmeyer 2006[50]**	**Study Design:** RCT, CT, BA<br>**Participants:** Providers<br>**Interventions: Skill mix - role expansion**, educational materials, skills and competencies development<br>**Search Dates:** Up to 2001<br>**Focus:** Effect of pharmacist care on treatment adherence	• 5 included studies (2 excluded from analysis)<br>**Glycemic Control (Process):**<br>• 1/1 study favoured intervention<br>**Glycemic Control (Clinical):**<br>• 3/3 studies favoured intervention (1/1 RCT)<br>**Retinopathy Screening:**<br>• 1/1 study favoured intervention<br>**Foot Screening:**<br>• 1/1 study favoured interventions	No meta-analysis or meta-regression.
**Loveman 2003[51]**	**Study Design:** RCT, CCT<br>**Participants:** Patients, Providers<br>**Interventions: Skill mix -role expansion**, other ICT that support individuals who provide care, behaviour change support<br>**Search Dates:** Up to 2002<br>**Focus:** Effect of diabetes specialist nurses	• 6 included studies (0 excluded from analysis)<br>**Glycemic Control (Clinical):**<br>• 4/6 studies favoured intervention (3/5 RCTs)	No meta-analysis or meta-regression.
**Machado 2007[54]**	**Study Design:** RCT, CT, BA, Other<br>**Participants:** Patients, Providers<br>**Interventions: Skill mix - role expansion**, information or education provision, behaviour change support, continuity of care, personal support<br>**Search Dates:** Up to 2006<br>**Focus:** Pharmacist-delivered interventions	• 36 included studies (5 excluded from analysis)<br>**Glycemic Control (Clinical):**<br>• 27/29 studies favoured intervention (13/14 RCTs)<br>**Vascular Risk Factors (Clinical):**<br>**Cholesterol:**<br>• 6/6 studies favoured intervention (2/2 RCTs)<br>**Blood Pressure:**	• Review authors' calculations of meta-analytic differences of the changes HbA1c from baseline to endpoint of intervention and control groups (n=14) revealed a statistically significant difference favouring the pharmacists' intervention group. The control group mean declined by 0.28%, but intervention reduced it further (0.62% ± 0.29%; p=0.03).

Lead Author, Year, Reference	Review Characteristics	Main Results†	Reported effect sizes and potential effect modifiers‡
		• 8/8 studies favoured intervention (4/4 RCTs)	
**Norris 2006[57]**	**Study Design:** RCT, BA, Other<br>**Participants:** Patients, System, Other<br>**Interventions: Skill mix - multidisciplinary teams, skill mix - role substitution**, personal support, behaviour change support, information or education provision<br>**Search Dates:** Up to March 2004<br>**Focus:** Effectiveness of community health workers	• 18 included studies (6 studies excluded from analysis)<br>**Glycemic Control (Process):**<br>• 1/1 RCT favoured intervention<br>**Glycemic Control (Clinical):**<br>• 8/8 studies favoured intervention (4/4 RCTs)<br>**Vascular Risk Factors (Clinical):**<br>**Cholesterol:**<br>• 1/1 study favoured intervention<br>**Blood Pressure:**<br>• 3/3 studies favoured intervention (2/2 RCTs)	No meta-analysis or meta-regression.
**Wubben 2008[74]**	**Study Design:** RCT, CBA<br>**Participants:** Patients, Providers<br>**Interventions: Skill mix -role expansion**, staff - shared decision making, skills and competencies development, behaviour change support<br>**Search Dates:** Up to August 2007<br>**Focus:** Interventions involving pharmacists	• 21 included studies (1 excluded from analysis)<br>**Glycemic Control (Clinical):**<br>• 15/18 studies favoured intervention (6/8 RCTs)<br>**Vascular Risk Factors (Clinical):**<br>**Cholesterol:**<br>• 8/10 studies favoured intervention (3/4 RCTs)<br>**Blood Pressure:**<br>• 9/10 studies favoured intervention (4/4 RCTs)	No meta-analysis or meta-regression.
**ORGANISATIONAL CHANGES INTERVENTIONS**			
**Al-Ansary 2011[24]**	**Study Design:** RCT<br>**Participants:** Patients, Providers	• 7 studies (4 excluded from analysis)	• Meta-analysis favours intervention for

Lead Author, Year, Reference	Review Characteristics	Main Results†	Reported effect sizes and potential effect modifiers‡
	**Interventions:** Integration of services<br>**Search Dates:** Up to Aug 2010<br>**Focus:** Point of care testing for HbA1c in the management of diabetes	**Glycemic Control (Clinical):**<br>• 3/3 RCTs favoured intervention	the 3 studies where data was reported change in HbA1c was reported in the trials (-0.09% (-0.21 to 0.02)).
**Clar 2007[32]**	**Study Design:** RCT, CT, CBA, Other<br>**Participants:** Patients, Providers, Other, System<br>**Interventions:** Site of service delivery<br>**Search Dates:** Up to November 2006<br>**Focus:** Out-patient or home-based management for children	• 7 included studies (3 excluded from analysis)<br>**Glycemic Control (Clinical):**<br>• 2/4 studies favoured intervention (2/2 RCTs)	No meta-analysis or meta-regression.
**Foy 2010[40]**	**Study Design:** BA<br>**Participants:** Providers<br>**Interventions: Staff – Shared decision making**, **case management**, ICT that support individuals who provide care<br>**Search Dates:** Up to June 2008<br>**Focus:** Interactive communication between collaborating primary care physicians and specialists	• 23 included studies (18 excluded from analysis)<br>**Glycemic Control (Clinical):**<br>• 5/5 studies favoured intervention	Pooled effect size for HbA1c levels was -0.64 (-0.93 to -0.34), which translates to an improvement of 1.4% in HbA1c in diabetic patients
**Griffin 1998[44]**	**Study Design:** RCT<br>**Participants:** Providers<br>**Interventions: Site of service delivery**, educational materials, reminders and prompts, behaviour change support<br>**Search Dates:** Not specified<br>**Focus:** General practice care	• 6 included studies (1 excluded from analysis)<br>**Glycemic Control (Clinical):**<br>• 3/4 RCTs favoured intervention	• Overall, there was no significant difference in five studies that evaluated metabolic control of patients receiving general practice and hospital care; the weighted difference in mean HbA1c was -0.005% (-0.26% to 0.25%).

Lead Author, Year, Reference	Review Characteristics	Main Results†	Reported effect sizes and potential effect modifiers‡
**BROAD BASED REVIEWS**			
**Chodosh 2005[31]**	**Study Design:** RCT<br>**Participants:** Patients, Providers, Other<br>**Interventions: Behaviour change support, information or education provision**, skill mix changes (role expansion, substitution or use of teams), skills and competencies development, culturally appropriate care<br>**Search Dates:** 1980 up to September 2004<br>**Focus:** Self-management for chronic diseases	• 53 included studies (34 excluded from analysis[∫])<br>**Glycemic Control (Clinical):**<br>• 16/19 RCTs favoured intervention	Twenty comparisons from 20 diabetes studies reported HbA1c outcomes. In an overall analysis of the effectiveness of chronic disease self-management programs, these studies reported a statistically and clinically significant pooled effect size of -0.36 (-0.52 to -0.21) in favour of the intervention.
**Glazier 2006[43]**	**Study Design:** RCT, CT, CBA<br>**Participants:** Patients, Providers, Other<br>**Interventions: Provider roles** (**skill mix - role expansion, skill mix - multidisciplinary teams**, educational materials, culturally appropriate care, information or education provision, behaviour change support), **Patient education** (**information or education provision**, behaviour change support, personal support, culturally appropriate care, skill mix - multidisciplinary teams), **Provider targeted** (reminders and prompts, other ICT that support individuals that provide care, information or education provision,	• 17 included studies (2 excluded from analysis)<br>**Glycemic Control (Clinical):**<br>• 6/6 studies favoured provider role interventions (2/2 RCTs)<br>• 4/4 favoured patient education interventions (3/3 RCTs)<br>• 1/1 study favoured provider targeted interventions<br>**Vascular risk factors (Clinical):**<br>**Cholesterol:**<br>• 2/2 studies favoured provider role interventions<br>**Blood Pressure:**<br>• 1/1 RCT favoured provider role interventions<br>• 1/1 study favoured patient education interventions	No meta-analysis or meta-regression.

Lead Author, Year, Reference	Review Characteristics	Main Results†	Reported effect sizes and potential effect modifiers‡
	behaviour change support)<br>**Search Dates:** January 1986 up to December 2004<br>**Focus:** Socially disadvantaged populations	**Retinopathy screening:**<br>• 2/2 studies favoured provider role interventions<br>• 1/1 RCT favoured patient education interventions<br>**Foot Screening:**<br>• 2/2 studies favoured provider role interventions<br>**Renal Function Monitoring:**<br>• 1/1 study favoured provider role interventions	
**Pimouguet 2011[61]**	**Study Design:** RCT<br>**Participants:** Patients, Provider<br>**Interventions: Education** (behaviour change support, information or education provisions, personal support), **Coaching** (personal support), **Monitoring** (patient mediated), **PCP Feedback** (behaviour change support, local consensus processes), **Treatment Adjustments** (skill mix – role expansion or extension)<br>**Search Dates:** Up to December 2009<br>**Focus:** Disease-management programs for improving diabetes care	• 41 included studies (0 excluded from analysis)<br>**Glycemic Control (Clinical)**<br>• 39/41 RCTs favoured interventions (2 showed no difference between control and intervention group)	• Disease management programs result in a significant reduction in HbA1c levels (pooled standardised mean difference between intervention and control groups -0.38 (-0.47 to -0.29), which corresponds to an absolute mean difference of 0.51%).<br>• Programs in which the disease manager was able to start or modify treatment with or without prior approval from the primary care physician resulted in a greater improvement in HbA1c levels (standardised mean difference -0.60 v. -0.28 in trials with no approval to do so; p<0.001).<br>• Programs with a moderate or high frequency of contact reported a significant reduction in HbA1c levels compared with usual care; nevertheless, only programs with a high frequency of contact led to a

Lead Author, Year, Reference	Review Characteristics	Main Results†	Reported effect sizes and potential effect modifiers‡
			significantly greater reduction compared with low frequency contact programs (SMD -0.56 vs. -0.30, p=0.03).
**Renders 2001[63]**	**Study Design:** RCT, CBA, ITS<br>**Participants:** Patients, Providers, Other, System<br>**Interventions: Provider targeted** (educational materials, educational meetings, educational outreach, audit and feedback, local consensus process, reminders and prompts), **Provider and Patient targeted** (educational materials, educational meetings, reminders and prompts, information or education provision), **System targeted** (skill mix - role expansion), **System and Patient targeted** (staff - continuity of care, skill mix - multidisciplinary teams, information or education provision), **Provider and System targeted** (educational materials, reminders and prompts, audit and feedback, health record systems, staff - continuity of care, educational meetings, skill mix - role expansion, skill mix - multidisciplinary team), **Provider, Patient and System targeted** (educational materials, local consensus processes, audit and feedback, patient mediated, skill	• 41 included studies (8 excluded from analysis)<br>**Glycemic Control (Process):**<br>• 4/4 RCTs favoured intervention for provider targeted interventions<br>• 2/2 studies favoured intervention for system targeted interventions<br>• 4/5 studies favoured intervention for provider and system targeted interventions (2/3 RCTs)<br>• 0/1 study favoured intervention for provider, patient and system targeted interventions<br>**Glycemic Control (Clinical):**<br>• 2/4 studies favoured intervention for provider targeted interventions (1/3 RCTs)<br>• 2/2 studies favoured intervention for provider and patient targeted interventions (1/1 RCT)<br>• 1/1 study favoured intervention for system targeted interventions<br>• 3/3 studies favoured intervention for system and patient targeted interventions (2/2 RCTs)<br>• 7/9 studies favoured intervention for provider and system targeted interventions (4/6 RCTs)<br>• 3/3 studies favoured intervention for provider, patient and system	No meta-analysis or meta-regression.

Lead Author, Year, Reference	Review Characteristics	Main Results†	Reported effect sizes and potential effect modifiers‡
	mix - role expansion, staff - continuity of care, physical structure, facilities and equipment, health record systems, information or education provision, ICT that support individuals who receive care)<br>**Search Dates:** Up to 2000<br>**Focus:** Professional and structural interventions to improve care	targeted interventions (2/2 RCTs)<br>**Vascular risk factors (Process):**<br>• 1/1 RCT favoured intervention for provider, patient and system targeted interventions<br>**Vascular risk factors (Clinical):**<br>**Cholesterol:**<br>• 2/2 studies favoured intervention for provider and patient targeted interventions (1/1 RCT)<br>• 1/1 RCT favoured intervention for system and patient targeted interventions<br>• 1/1 study favoured intervention for provider and system targeted interventions<br>• 1/2 RCTs favoured intervention for provider, patient and system targeted interventions<br>**Blood Pressure:**<br>• 0/1 RCT favoured intervention for provider targeted interventions<br>• 1/2 studies favoured intervention for provider and patient targeted interventions (0/1 RCT)<br>• 1/1 study had mixed effects for system and patient targeted interventions<br>• 0/2 RCTs favoured intervention for provider and system targeted interventions<br>• 0/2 RCTs favoured intervention for provider, patient and system targeted interventions	

Lead Author, Year, Reference	Review Characteristics	Main Results†	Reported effect sizes and potential effect modifiers‡
		**Retinopathy Screening:**<br>• 5/6 studies favoured intervention for provider targeted interventions (5/5 RCTs)<br>• 2/2 studies favoured intervention for system targeted interventions<br>• 1/1 RCT favoured intervention for system and patient targeted interventions<br>• 4/5 studies favoured intervention for provider and system targeted interventions (1/1 RCT)<br>• 1/1 RCT favoured intervention for provider, patient and system targeted interventions<br>**Foot Screening:**<br>• 3/3 RCTs favoured intervention for provider targeted interventions<br>• 1/1 study favoured intervention for provider and patient targeted interventions<br>• 1/1 study favoured intervention for system targeted interventions<br>• 4/4 studies favoured intervention for provider and system targeted interventions (1/1 RCT)<br>• 1/1 RCT favoured intervention for provider, patient and system targeted interventions<br>**Renal Function Monitoring:**<br>• 2/3 studies favoured intervention for provider targeted interventions (2/2 RCTs)<br>• 0/1 study had mixed results for	

Lead Author, Year, Reference	Review Characteristics	Main Results†	Reported effect sizes and potential effect modifiers‡
		system targeted interventions<br>• 2/2 studies favoured intervention for provider and system targeted interventions (1/1 RCT)<br>• 2/2 RCTs favoured intervention for provider, patient and system targeted interventions	
**Saxena 2007[66]**	**Study Design:** RCT, CT<br>**Participants:** Providers, System, Other<br>**Interventions: Case management by specialist nurse or link worker** (skill mix - multidisciplinary teams, substitution and role expansion, educational meetings, educational materials, culturally appropriate care, information or education provision)<br>**Search Dates:** Up to December 2006<br>**Focus:** Minority ethnic groups	• 9 included studies (1 excluded from analysis)<br>**Glycemic Control (Clinical):**<br>• 6/7 studies favoured intervention for case management interventions (5/6 RCTs)<br>**Vascular risk factors (Clinical):**<br>**Cholesterol:**<br>• 4/5 RCTs favoured intervention for case management interventions<br>**Blood Pressure:**<br>• 3/5 RCTs favoured intervention for case management interventions	No meta-analysis or meta-regression.
**Shojania 2006 [16]**<br>**(**companion paper)[17]	**Study Design:** RCT, CBA, CT<br>**Participants:** Patients, Providers, Other (not-specified)<br>**Interventions: Patient and System targeted** (**information or education provision**, behaviour change support, skill mix - role expansion and multidisciplinary teams, site of service delivery, other ICT that supports individuals who provide care), **Provider and System targeted** (skill mix - role expansion, educational meetings, patient-mediated, reminders and	• 58 included studies (30 excluded from analysis)<br>**Glycemic Control (Process):**<br>• 2/3 RCTs favoured intervention for provider and system targeted interventions<br>• 0/1 RCT favoured intervention for patient, provider and system targeted interventions<br>• 0/1 RCT favoured intervention for provider targeted interventions<br>**Glycemic Control (Clinical):**<br>• 7/9 studies favoured intervention for patient and system targeted	• Pooled analysis of 66 studies revealed that interventions reduced HbA1c values by a mean of 0.42% over a median of 13 months of follow-up. Team changes and case management quality improvement strategies were the only interventions associated with significant reductions in HbA1c values (at least 0.50%).<br>• Patient education, which was present in 38 trials, was associated with an incremental reduction in HbA1c values of 0.15% (p=0.20); patient reminders, present in 14 trials, were

Lead Author, Year, Reference	Review Characteristics	Main Results†	Reported effect sizes and potential effect modifiers‡
	prompts, educational materials, other ICT that supports individuals who provide care, ICT that supports individuals who receive care, staff - continuity of care), **Patient, Provider and System targeted** (**skill mix - role expansion, behaviour change support**, patient-mediated, educational meetings, other ICT that supports individuals who provide care, reminders and prompts, information or education provision, educational materials, audit and feedback, staff continuity of care, staff - shared decision making), **Provider targeted** (**reminders and prompts**, educational materials, educational meetings, patient-mediated, local opinion leaders, information or education provision, behaviour change support), **Provider and Patient targeted** (**reminders and prompts educational materials**, educational meetings, patient-mediated, local opinion leaders, information or education provision, behaviour change support), **Patient targeted** (information or education provision, behaviour change support), **System targeted** (other ICT that supports individuals who provide care, skill mix - role	interventions (5/7 RCTs)<br>• 5/5 RCTs favoured intervention for provider and system targeted interventions<br>• 6/6 studies favoured intervention for patient, provider and system targeted interventions (2/2 RCTs)<br>• 2/3 studies favoured intervention for provider targeted interventions (1/2 RCTs)<br>• 3/3 RCTs favoured intervention for patient and provider targeted interventions<br>• 2/2 RCTs favoured intervention for patient targeted interventions<br>• 1/2 RCTs favoured intervention for system targeted interventions<br>**Vascular risk factors (Clinical):**<br>**Blood Pressure (Systolic)**<br>• 1/2 studies favoured intervention for patient and system targeted interventions (1/1 RCT)<br>• 2/3 RCTs favoured intervention for provider and system targeted interventions<br>• 0/1 RCT favoured intervention for patient, provieder and system targeted interventions<br>• 1/2 RCTs favoured intervention for provider targeted interventions<br>• 1/1 RCT favoured intervention for patient and provider targeted interventions<br>**Blood Pressure (Diastolic):**	associated with an incremental reduction of 0.11% (p=0.40).<br>• Interventions in which nurse or pharmacist case managers could make medication adjustments without awaiting physician authorization reduced values more than all other interventions.

Lead Author, Year, Reference	Review Characteristics	Main Results†	Reported effect sizes and potential effect modifiers‡
	expansion, staff - continuity of care)<br>**Search Dates:** Up to April 2006<br>**Focus:** Quality improvement strategies	• 2/2 studies favoured intervention for patient and system targeted interventions (1/1 RCT)<br>• 2/3 RCTs favoured intervention for provider and system targeted interventions<br>• 0/1 RCT favoured intervention for patient, provider and system targeted interventions<br>• 2/2 RCTs favoured intervention for provider targeted interventions<br>• 1/1 RCT favoured intervention for provider and patient interventions<br>**Retinopathy Screening:**<br>• 1/1 RCT favoured intervention for patient and system interventions<br>• 1/1 RCT favoured intervention for provider and system interventions<br>**Foot Screening:**<br>• 1/1 RCT favoured intervention for patient and system interventions<br>• 1/1 RCT favoured intervention for provider and system interventions	
**Vermeire 2005[71]**	**Study Design:** RCT, CT, CBA, Other<br>**Participants:** Patients, Providers, Other<br>**Interventions:** Skill mix - role expansion (nurse), skill mix - role expansion (pharmacist), information or education provision, skills and competencies development, behaviour change support	• 21 included studies (10 excluded from analysis)<br>**Glycemic Control (Clinical):**<br>• 2/2 RCTs favoured intervention for skill mix - role expansion (nurse-led care) interventions<br>• 3/4 studies favoured intervention for skill mix - role expansion (pharmacist-led care) interventions (1/1 RCT)<br>• 1/2 studies favoured intervention	• The review authors pooled the studies assessing educational interventions: those facilitating adherence and those offering diabetes education versus usual care or a control. Pharmacist-led care was associated with a mean difference in HbA1c levels of -0.71 (-1.24 to -0.17) (n=4). Nurse-led care was associated with a mean difference in HbA1c levels of -0.10 (-0.12 to -0.08) (n=2).

Lead Author, Year, Reference	Review Characteristics	Main Results†	Reported effect sizes and potential effect modifiers‡
	**Search Dates:** Up to January 2002<br>**Focus:** Adherence to treatment recommendations	for information or education provision<br>• 1/1 RCT favoured intervention for skills and competencies development<br>• 1/1 RCT favoured intervention for behaviour change support<br>**Vascular risk factors (Clinical):**<br>**Cholesterol:**<br>• 0/1 RCT favoured intervention for skills and competencies development<br>**Blood Pressure:**<br>• 1/1 study favoured intervention for information or education provision<br>• 0/1 RCT favoured intervention for skills and competencies development<br>**Smoking Cessation (Clinical):**<br>• 1/1 RCT favoured intervention for skill mix - role expansion (nurse-led care)<br>• 1/1 RCT favoured intervention for information or education provision	
**Zhang 2007[75]**	**Study Design:** RCT, CT, CBA, BA<br>**Participants:** Patients, Providers, Other, System<br>**Interventions:** Reminders and prompts, ICT that supports individuals who provide care/electronic health records, site of service delivery, educational meetings, skill mix – multidisciplinary teams/skill mix –	• 48 included studies (8 excluded from analysis)<br>**Retinopathy Screening:**<br>• 9/9 studies favoured intervention for reminders and prompts (1/1 RCT)<br>• 9/10 studies favoured intervention for ICT that supports individuals who provide care/electronic health records (5/5 RCTs)	No meta-analysis or meta-regression.

Lead Author, Year, Reference	Review Characteristics	Main Results†	Reported effect sizes and potential effect modifiers‡
	role expansion/staff – continuity of care, package of care/scaling-up coverage<br>**Search Dates:** Up to May 2005<br>**Focus:** Retinal screening	• 5/5 studies favoured intervention for site of service delivery interventions (1/1 RCT)<br>• 10/11 studies favoured intervention for educational meetings<br>• 3/3 studies favoured intervention for skill mix and staff interventions<br>• 2/2 studies favoured intervention for package of care/scaling-up coverage interventions	

Key: RCT = Randomised Control Trial, CT = Control Trial, CBA = Controlled Before and After, BA = Before and After, ITS = Interrupted Time Series , CCT = Clustered Randomised Control Trial, COT = Cross Over Trial, ICT = Information or Communication Technology, SMBG = Self-Monitored Blood Glucose, BP = Blood Pressure, SBP = Systolic Blood Pressure, DBP = Diastolic Blood Pressure, SMD = Standardised Mean Difference, WMD = Weighted Mean Difference, MD = Mean Difference, HbA1c = Glycosylated Hemoglobin A_{1c} (measure of blood glucose)

† Based on vote-counting using direction of effect

‡ Reported effect sizes and potential effect modifiers are those reported by the review authors. The number of studies included for a given outcome in a given meta-analysis may differ from the number of studies included in the overview as we excluded some studies due to interventions deemed not relevant to the overview project.

∫ 27 included studies not related to diabetes and were excluded from our analysis

a Meta-analyses conducted by Valk *et al* included only one study; the results are not reported here.

Number of included studies is the total number of studies included by the review authors.

Where interventions were classified as being complex or multifaceted, the main intervention used in the majority of studies is highlighted in bold font. When there is only one outcome reported, the main results are presented for that outcome by intervention. When there is only one intervention (or multifaceted is specified), the main results are presented without specifying the intervention. Where there are multiple outcomes and multiple interventions, the main results are grouped by outcome domain and reported by intervention.
