# Supplementary material for: Improving quality of care for persons with diabetes: an overview of systematic reviews - what does the evidence tell us?
Source: Syst Rev. 2013 May 7;2:26. doi: 10.1186/2046-4053-2-26 (PMC3667096; doi:10.1186/2046-4053-2-26)
Supplement: Additional file 4 — List of excluded reviews. Bibliography of excluded reviews (reviews that scored less than 5 using AMSTAR tool). [file 2046-4053-2-26-S4.pdf]

Additional file 4 - List of excluded reviews

Number	Citation
1	Albano MG, Crozet C, D'Ivernois JF. Analysis of the 2004-2007 literature on therapeutic patient education in diabetes: Results and trends. *Acta Diabetol* 2008; 45(4): 211-219.
2	Armour TA, Norris SL, Jack J, Zhang X, Fisher L. The effectiveness of family interventions in people with diabetes mellitus: A systematic review. *Diabet Med* 2005; 22(10): 1295-1305.
3	Azar M, Gabbay R. Web-based management of diabetes through glucose uploads: has the time come for telemedicine? *Diabetes Res Clin Pract* 2009; 83(1): 9-17.
4	Balas EA, Boren SA, Griffing G. Computerized management of diabetes: a synthesis of controlled trials. *Proc AMIA Symp*. 1998: 295-299
5	Baradaran HR, Shams-Hosseini N, Noori-Hekmat S, Tehrani-Banihashemi A, Khamseh ME. Effectiveness of diabetes educational interventions in Iran: a systematic review. *Diabetes Technol Ther* 2010; 12(4): 317-331.
6	Bazian Ltd. Education to prevent foot ulcers in diabetes. *Evid Base Healthc Publ Health* 2005; 9(5): 351-358.
7	Blenkinsopp A, Hassey A. Effectiveness and acceptability of community pharmacy-based interventions in type 2 diabetes: A critical review of intervention design, pharmacist and patient perspectives. *Int J Pharm Pract* 2005; 13(4): 231-240.
8	Boren SA, Fitzner KA, Panhalkar PS, Specker JE. Costs and benefits associated with diabetes education: a review of the literature. *Diabetes Educ* 2009; 35(1): 72-96.
9	Boren SA, Gunlock TL, Peeples MM, Krishna S. Computerized learning technologies for diabetes: a systematic review. *J Diabetes Sci Technol* 2008; 2(1): 139-146.
10	Boren SA, Puchbauer AM, Williams F. Computerized prompting and feedback of diabetes care: a review of the literature. *J Diabetes Sci Technol* 2009; 3(4): 944-950.
11	Boren SA. A review of health literacy and diabetes: opportunities for technology. *J Diabetes Sci Technol* 2009; 3(1): 202-209.
12	Conn VS, Hafdahl AR, Mehr DR, Lemaster JW, Brown SA, Nielsen PJ. Metabolic effects of interventions to increase exercise in adults with type 2 diabetes. *Diabetologia* 2007; 50(5): 913-921.
13	DeCoster VA, Cummings SM. Helping adults with diabetes: a review of evidence-based interventions. *Health Soc Work* 2005; 30(3): 259-264.
14	DeCoster VA. Diabetes treatments. *J Gerontol Soc Work* 2008; 50(1): 105-129.
15	Eakin EG, Bull SS, Glasgow RE, Mason M. Reaching those most in need: a review of diabetes self-management interventions in disadvantaged populations. Diabetes *Metab Res Rev* 2002; 18(1): 26-35.
16	Eastridge DK. An integrative review of interventions to reduce peripheral arterial disease risk factors in African Americans. *J Vasc Nurs* 2009; 27(2): 31-45.
17	Eslami S, Abu-Hanna A, de Jonge E, de Keizer NF. Tight glycemic control and computerized decision-support systems: a systematic review. *Intensive Care Med* 2009; 35(9): 1505-1517.
18	Faas A, Schellevis FG, Van Eijk JT. The efficacy of self-monitoring of blood glucose in NIDDM subjects. A criteria-based literature review. *Diabetes Care* 1997; 20(9): 1482-1486.
19	Fan L, Sidani S. Effectiveness of diabetes self-management education intervention elements: A meta-analysis. *Can J Diabetes* 2009; 33(1): 18-26.
20	Gialamas A, St JA, Laurence CO, Bubner TK, PoCT Management Committee. Point-of-care testing for patients with diabetes, hyperlipidaemia or coagulation disorders in the general practice setting: a systematic review. *Fam Pract* 2010; 27(1): 17-24.

21	Graziano JA, Gross CR. The effects of isolated telephone interventions on glycemic control in type 2 diabetes: a literature review. *Adv Nurs Sci* 2009; 32(3): E28-E41.
22	Greenhalgh PM. Shared care for diabetes. A systematic review. *Occas Pap R Coll Gen Pract* 1994; 67: i-viii, 1-35.
23	Guldberg TL, Lauritzen T, Kristensen JK, Vedsted P. The effect of feedback to general practitioners on quality of care for people with type 2 diabetes. A systematic review of the literature. *BMC Fam Pract* 2009; 10: 30.
24	Harris M, Smith B, Veale A. Printed patient education interventions to facilitate shared management of chronic disease: A literature review. *Intern Med J* 2005; 35(12): 711-716.
25	Harris SB, Petrella RJ, Leadbetter W. Lifestyle interventions for type 2 diabetes. Relevance for clinical practice. *Can Fam Physician* 2003; 49(Dec): 1618-1625.
26	Hatherly K, Overland J, Smith L, Taylor S, Johnston C. Providing optimal service delivery for children and adolescents with type 1 diabetes: A systematic review. *Pract Diabetes Int* 2009; 26(4):154-159.<br>Link: DO - http://dx.doi.org/10.1002/pdi.1360.
27	Hill-Briggs F, Gemmell L. Problem solving in diabetes self-management and control: a systematic review of the literature. *Diabetes Educ* 2007; 33(6): 1032-1050.
28	Jaana M, Pare G. Home telemonitoring of patients with diabetes: a systematic assessment of observed effects. *J Eval Clin Pract* 2007; 13(2): 242-53.
29	Jack J. Diabetes self-management education research: An international review of intervention methods, theories, community partnerships and outcomes. *Dis Manag Health Out* 2003; 11(7): 415-428.
30	Jackson CL, Bolen S, Brancati FL, Batts-Turner ML, Gary TL. A systematic review of interactive computer-assisted technology in diabetes care. Interactive information technology in diabetes care. *J Gen Intern Med* 2006; 21(2): 105-110.
31	Jackson L. Translating the Diabetes Prevention Program into practice: a review of community interventions. *Diabetes Educ* 2009; 35(2): 309-320.
32	Khunti K, Camosso-Stefinovic J, Carey M, Davies MJ, Stone MA. Educational interventions for migrant South Asians with Type 2 diabetes: A systematic review. *Diabet Med* 2008; 25(8): 985-992.
33	Knight K, Badamgarav E, Henning JM, Hasselblad V, Gano AD, Jr., Ofman JJ, et al. A systematic review of diabetes disease management programs. *Am J Manag Care* 2005; 11(4): 242-250.
34	Krishna S, Boren SA. Diabetes Self-Management Care via Cell Phone: A Systematic Review. *J Diabetes Sci Technol* 2008; 2(3): 509-517.
35	Lacey KO, Chyun DA, Grey M. An integrative literature review of cardiac risk factor management in diabetes education interventions. *Diabetes Educ* 2000; 26(5): 812-820.
36	Leykum LK, Pugh J, Lawrence V, Parchman M, Noel PH, Cornell J, et al. Organizational interventions employing principles of complexity science have improved outcomes for patients with Type II diabetes. *Implement Sci* 2007; 2(28): doi:10.1186/1748-5908-2-28.
37	Mason J, O'Keeffe C, McIntosh A, Hutchinson A, Booth A, Young RJ. A systematic review of foot ulcer in patients with type 2 diabetes mellitus. I: prevention. *Diabet Med* 1999; 16(10): 801-812.
38	Mc Manus V, Savage E. Cultural perspectives of interventions for managing diabetes and asthma in children and adolescents from ethnic minority groups. *Child Care Health Dev* 2010; 36(5): 612-622.
39	McAndrew L, Schneider SH, Burns E, Leventhal H. Does patient blood glucose monitoring improve diabetes control? A systematic review of the literature. *Diabetes Educ* 2007; 33(6): 991-1011.
40	McBroom LA, Enriquez M. Review of family-centered interventions to enhance the health

	outcomes of children with type 1 diabetes. *Diabetes Educ* 2009; 35(3): 428-438.
41	Montani S, Bellazzi R, Quaglini S, d'Annunzio G. Meta-analysis of the effect of the use of computer-based systems on the metabolic control of patients with diabetes mellitus. *Diabetes Technol Ther* 2001; 3(3): 347-356.
42	Munro N, Felton A, McIntosh C. Is multidisciplinary learning effective among those caring for people with diabetes? *Diabet Med* 2002; 19(10): 799-803.
43	Murphy HR, Rayman G, Skinner TC. Psycho-educational interventions for children and young people with Type 1 diabetes. *Diabet Med* 2006; 23(9): 935-943.
44	Naik AD, Issac TT, Street J, Kunik ME. Understanding the quality chasm for hypertension control in diabetes: A structured review of "co-maneuvers" used in clinical trials. *J Am Board Fam Med* 2007; 20(5): 469-478.
45	Nakhla M, Daneman D, Frank M, Guttmann A. Translating transition: a critical review of the diabetes literature. *J Pediatr Endocrinol Metab* 2008; 21(6): 507-516.
46	Newman S, Steed L, Mulligan K. Self-management interventions for chronic illness. *Lancet* 2004; 364(9444): 1523-1537.
47	Nichols PJ, Norris SL. A systematic literature review of the effectiveness of diabetes education of school personnel. *Diabetes Educ* 2002; 28(3): 405-414.
48	Norris SL, Lau J, Smith SJ, Schmid CH, Engelgau MM. Self-management education for adults with type 2 diabetes: a meta-analysis of the effect on glycemic control. *Diabetes Care* 2002; 25(7): 1159-71.
49	Norris SL, Nichols PJ, Caspersen CJ, Glasgow RE, Engelgau MM, Jack L, et al. The effectiveness of disease and case management for people with diabetes. A systematic review. *Am J Prev Med* 2002; 22(4 Suppl): 15-38.
50	O'Brien T, Denham SA. Diabetes care and education in rural regions. *Diabetes Educ* 2008; 34(2): 334-347.
51	O'Mullane M, McHugh S, Bradley CP. Informing the development of a national diabetes register in Ireland: a literature review of the impact of patient registration on diabetes care. *Inform Prim Care* 2010; 18(3): 157-168.
52	Osborn CY, Mayberry LS, Mulvaney SA, Hess R. Patient web portals to improve diabetes outcomes: a systematic review. *Curr Diab Rep* 2010; 10(6): 422-435.
53	Padgett D, Mumford E, Hynes M, Carter R. Meta-analysis of the effects of educational and psychosocial interventions on management of diabetes mellitus. *J Clin Epidemiol* 1988; 41(10): 1007-1030.
54	Pare G, Moqadem K, Pineau G, St-Hilaire C. Clinical effects of home telemonitoring in the context of diabetes, asthma, heart failure and hypertension: a systematic review. *J Med Internet Res 2010*; 12(2): e21.<br>Link: doi 10.2196/jmir.1357
55	Peek ME, Cargill A, Huang ES. Diabetes health disparities: a systematic review of health care interventions. *Med Care Res Rev* 2007; 64(5 Suppl): 101S-156S.
56	Post PN, Wittenberg J, Burgers JS. Do specialized centers and specialists produce better outcomes for patients with chronic diseases than primary care generalists? A systematic review. *Int J Qual Health Care* 2009; 21(6): 387-396.
57	Ramadas A, Quek KF, Chan CKY, Oldenburg B. Web-based interventions for the management of type 2 diabetes mellitus: A systematic review of recent evidence. *Int J Med Inform* 2011; 80(6):389-405. Link: DO - http://dx.doi.org/10.1016/j.ijmedinf.2011.02.002.
58	Riley SB, Marshall ES. Group visits in diabetes care: a systematic review. *Diabetes Educ* 2010; 36(6): 936-944.
59	Ruiz-Aragon J, Portero RV, Moreno SF. Out-patient determination of glycosylated haemoglobin in the monitoring and control of diabetes mellitus: Systematic review of the literature. *Aten Primaria* 2008; 40(2): 69-74.

60	Saudek CD, Derr RL, Kalyani RR. Assessing glycemia in diabetes using self-monitoring blood glucose and hemoglobin A1c. *J Am Med Assoc* 2006; 295(14): 1688-1697.
61	Seitz P, Rosemann T, Gensichen J, Huber CA. Interventions in primary care to improve cardiovascular risk factors and glycated haemoglobin (HbA1c) levels in patients with diabetes: a systematic review. *Diabetes Obes Metab* 2011; 13(6): 479-489. Link: DO - http://dx.doi.org/10.1111/j.1463-1326.2010.01347.x.
62	Sigurdardottir AK, Jonsdottir H, Benediktsson R. Outcomes of educational interventions in type 2 diabetes: WEKA data-mining analysis. *Patient Educ Couns* 2007; 67(1-2): 21-31.
63	Sigurdardottir AK. Self-care in diabetes: model of factors affecting self-care. *J Clin Nurs* 2005; 14(3): 301-314.
64	Soo H, Lam S. Stress management training in diabetes mellitus. *J Health Psychol* 2009; 14(7): 933-943.
65	Srinivasan B, Taub N, Khunti K, Davies M. Diabetes: glycaemic control in type 2. *Clin Evid* 2008; 2008(3): 609-637.
66	Steed L, Cooke D, Newman S. A systematic review of psychosocial outcomes following education, self-management and psychological interventions in diabetes mellitus. *Patient Educ Couns* 2003; 51(1): 5-15.
67	Tolbert R. Managing type 1 diabetes at school: an integrative review. *J Sch Nurs* 2009; 25(1): 55-61.
68	Urban AD, Berry D, Grey M. Optimizing outcomes in adolescents with type 1 diabetes and their families. *J Clin Outcomes Manag* 2004; 11(5): 299-306.
69	van Bruggen JA, Gorter KJ, Stolk RP, Rutten GE. Shared and delegated systems are not quick remedies for improving diabetes care: a systematic review. *Prim Care Diabetes* 2007; 1(2): 59-68.
70	van Dam HA, van der Horst F, van den Borne B, Ryckman R, Crebolder H. Provider-patient interaction in diabetes care: effects on patient self-care and outcomes. A systematic review. *Patient Educ Couns* 2003; 51(1): 17-28.
71	Van Scoyoc EE, DeWalt DA. Interventions to improve diabetes outcomes for people with low literacy and numeracy: A systematic literature review. *Diabetes Spectr* 2010; 23(4): 228-237. Link: DO - http://dx.doi.org/10.2337/diaspect.23.4.228.
72	Verhoeven F, Tanja-Dijkstra K, Nijland N, Eysenbach G, van Gemert-Pijnen L. Asynchronous and synchronous teleconsultation for diabetes care: a systematic literature review. *J Diabetes Sci Technol* 2010; 4(3): 666-684.
73	Warsi A, Wang PS, LaValley MP, Avorn J, Solomon DH. Self-management education programs in chronic disease: A systematic review and methodological critique of the literature. *AMA Arch Intern Med* 2004; 164(15): 1641-1649.
74	Whittemore R. Culturally competent interventions for Hispanic adults with type 2 diabetes: a systematic review. *J Transcult Nurs* 2007;18(2):157-166.
75	Whittemore R. Strategies to facilitate lifestyle change associated with diabetes mellitus. *J Nurs Scholarsh* 2000; 32(3): 225-232.
